# Comparing photosynthetic light harvesting of single photons and pseudothermal light under ultraweak illumination

**DOI:** 10.1126/sciadv.adz2616

**Published:** 2025-11-14

**Authors:** Quanwei Li, Liwen Ko, K. Birgitta Whaley, Graham R. Fleming

**Affiliations:** ^1^ Department of Chemistry, University of California, Berkeley, Berkeley, CA 94720, USA.; ^2^ Kavli Energy Nanoscience Institute at Berkeley, Berkeley, CA 94720, USA.; ^3^ Molecular Biophysics and Integrated Bioimaging Division, Lawrence Berkeley National Laboratory, Berkeley, CA 94720, USA.

## Abstract

Photosynthesis in vivo is driven by sunlight, an ultraweak incoherent thermal source. However, most experiments and theories have studied photosynthetic light harvesting driven by strong coherent laser sources. The quantum states of light are characterized by their photon statistics, in addition to classical properties such as intensity and frequency spectrum. Here, we report experiments that investigate how photon statistics affect a natural photosynthetic system and vice versa. We directly compare how single photons and pseudothermal light from spontaneous parametric down-conversion drive light harvesting in the light-harvesting 2 complex from a purple bacterium. We find that the fluorescence lifetime and quantum efficiency are unchanged while the fluorescence photon statistics are markedly different, resembling that of the incident light, implying that the dynamics do not fundamentally modify the photon statistics. This represents a step toward clarification of the similarities and differences between photosynthetic light harvesting in laboratory and in natural sunlight conditions.

## Teaser

Similarities and differences between photosynthetic light harvesting in laboratory and natural settings.

## INTRODUCTION

**Fig. 1.**
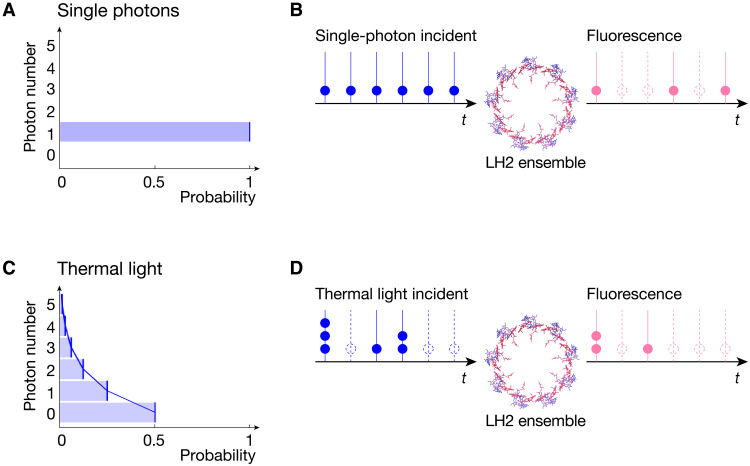
Principle of the experiments. (**A** and **C**) Photon statistics of single photons and thermal light, respectively, as described by their photon number distributions. The two different quantum states of light have all the same classical properties of light, including intensity, frequency spectrum, polarization, etc. (A) An ideal single-photon state contains exactly one photon with unit probability. In contrast, (C) a thermal state with average photon number equal to one is a mixed state with photon number ranging from zero to infinity in a Bose-Einstein probability distribution. (**B** and **D**) Schematic of a stream of light pulses incident onto an ensemble of LH2 complex from purple bacterium *R. sphaeroides*, in which each pulse is (B) a single photon and (D) thermal light with average photon number equal to one. After absorption of the light and multiple energy transfers within LH2, the fluorescence emission is detected and analyzed to study the effects of photon statistics of the incident light on the photosynthetic light harvesting. Blue and red solid dots represent incident and fluorescent photons, respectively. Dashed circles represent zero photons. The LH2 structure is produced from the Protein Data Bank file 1NKZ using ChimeraX, where the B800 ring (with 9 bacteriochlorophylls) and the B850 ring (with 18 bacteriochlorophylls) are shown in blue and red, respectively, and the carotenoids and proteins are not shown for simplicity.

At the fundamental quantum level, light is described in terms of photons—the smallest packets of light energy that cannot be further divided (*1*). The quantum nature of light introduces a new property, namely, the photon statistics, which characterize and distinguish different quantum states of light, even when all their classical properties, such as intensity, spectra, spatial mode, temporal mode, and polarization, are equal. For example, a single-photon state contains exactly one photon, whereas a thermal state is a mixture of different photon numbers ranging from zero to infinity, which follows a Bose-Einstein distribution specified by an average photon number (Fig. 1, A and C). In practice, photon statistics are usually quantified and measured by the second-order quantum coherence function of the light, *g*^(2)^(τ) (*2*–*5*), which is the joint probability of detecting two photons separated by time τ. For instance, at zero time delay, *g*^(2)^(τ = 0) = 0 for ideal single photons, and *g*^(2)^(τ = 0) = 2 for thermal light (*4*, *5*). Photon statistics described by *g*^(2)^(τ) contain quantum dynamical information about both the photon stream and the light source (*4*, *5*).

Sunlight is weak thermal light that drives natural photosynthesis, a vital process during which light energy is absorbed and converted into chemical energy, whose initial light reactions under low light level proceed with a remarkable near-unity quantum efficiency, to sustain life on Earth (*6*). Much effort has been invested in the elucidation of the microscopic mechanism of this crucial process, yet over the past several decades, most experiments and theories probing the microscopic dynamics of photosynthetic light harvesting have been restricted to studies using strong coherent laser light (*7*–*20*). Coherent laser light sources are usually much more intense and have fundamentally different photon statistics from sunlight [for lasers, the photon number follows a Poisson distribution with *g*^(2)^(τ) = 1 for all values of τ]. The question of whether thermal light and coherent laser light give rise to different behaviors in photosynthetic light harvesting has generated considerable discussion in recent years (*21*–*23*). However, all previous studies have only described the interaction of a photosynthetic system with light in terms of the first-order coherence [*g*^(1)^(τ)] and not considered whether or how light harvesting could be affected by the higher-order quantum coherences encoded in photon statistics.

Here, we experimentally investigate the effects of photon statistics of the incident light on photosynthetic light harvesting, to better understand the differences between laboratory studies of photosynthetic light harvesting using lasers and these same processes in pseudothermal conditions that are closer to the natural conditions under sunlight. Using time-resolved photon-counting quantum light spectroscopy (QLS) (*24*), we used single photons and pseudothermal light to drive photosynthetic light harvesting by an ensemble of light-harvesting 2 (LH2) complexes from the purple bacterium *Rhodobacter sphaeroides* under ambient conditions, using the same experimental setup in both cases (Fig. 1, B and D). After light absorption and energy transfer in LH2, we detected and analyzed the fluorescence emission signal to determine how the photon statistics of the incident light affect the lifetime, efficiency, and the photon statistics of the fluorescence.

## RESULTS

### The experimental setup

**Fig. 2.**
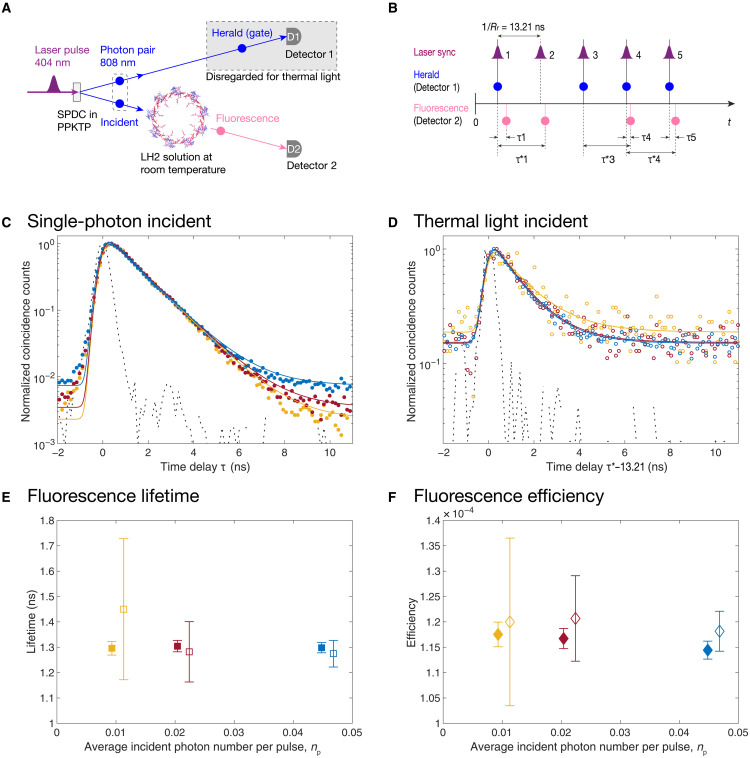
Fluorescence lifetime and quantum efficiency are the same for both incident photon statistics. (**A**) Simplified schematic of the experiments using time-resolved QLS. SPDC, spontaneous parametric down-conversion; PPKTP, periodically poled potassium titanyl phosphate. (**B**) Schematic of the raw time-tagged signal. The laser pulse synchronization signal was not recorded but is shown here as a guide. (**C** and **D**) Normalized coincidence counts between the two channels constructed from the histograms of their relative time differences shown in (B) with 128 ps bin size. In [(C) and (D)], the solid dots (open circles) are the measured data, shown colored to correspond to the three values of average photon number per pulse, *n*_p_, i.e., 0.0458 (rate 1, blue), 0.0214 (rate 2, red), and 0.0103 (rate 3, orange), respectively, with 2000-s integration time. The solid lines with the same color code are single exponential fits after reconvolution with instrument response functions (black dashed line) represented by the coincidence counts between heralds and incident residue (Materials and Methods and tables S2 to S4). (**E**) The fluorescence lifetime for incident single photons (solid squares) and thermal light (open squares) at the three different intensities, extracted from fitting of the data in [(C) and (D)]. (**F**) The overall efficiency of the fluorescence detection, for incident single photons (solid diamonds) and thermal light (open diamonds) at the three different intensities, extracted from fitting of the data in [(C) and (D)]. In [(E) and (F)], the three rates use the same color code as in [(C) and (D)], the error bars represent 95% confidence intervals of the fittings, and the data points are shifted −0.001 and +0.001 along the *x* axis for single photons and for thermal light, respectively, for clarity.

Our time-resolved photon-counting QLS (*24*) uses a spontaneous parametric down-conversion (SPDC) source (*25*). We generate single photons (*26*, *27*) and pseudothermal light (*28*, *29*) with different absolute intensities but otherwise identical classical properties (such as spectral distribution, polarization, temporal mode, and spatial mode) in the same setup under different measurement conditions (Fig. 2A). Laser pulses centered at 404 nm with repetition rate *R*_r_ = 75.7 MHz pump a nonlinear crystal to generate pulsed squeezed vacuum states around 808 nm in two separable spatial arms of a type II SPDC source. In each pulsed squeezed vacuum state, the two spatial modes in the two arms of the output light are entangled in photon number, with the light in both arms following the same thermal state photon number distribution (*28*). The upper arm is directed to detector 1 and may serve as a herald and a timed gate for the lower arm, which is incident onto the LH2 complex ensemble in solution at room temperature. The incident light pulse can become pseudothermal light when the upper arm is ignored (i.e., tracing out the upper arm), which makes the state of the incident arm to be a mixed state with a thermal photon number distribution. We shall refer to this as the “pseudothermal” light to acknowledge its origin from a nonlinear optical source rather than from sunlight (*30*). Such pseudothermal light is quantum mechanically identical to a real thermal light if it were to have the same spectral shape, temporal mode, polarization, and average intensity as a true thermal source such as sunlight. Alternatively, the incident light pulse can become a single photon with good approximation when conditioned on heralding by photon detection at detector 1 in the upper arm, because herald detection at detector 1 collapses the squeezed vacuum state and projects the incident pulse to be approximately a single photon (*26*, *27*). The choice of pseudothermal light or single photons can be delayed after all the data are recorded and then imposed in postprocessing. The average photon number per pulsed squeezed vacuum state, *n*_p_, is set to be 0.0458 (denoted as rate 1), 0.0214 (rate 2), and 0.0103 (rate 3) in the current experiments. These light intensities are estimated to be similar to that of natural sunlight at similar bandwidth under light-limited photosynthetic conditions (*31*). Specifically, the photon flux in a bandwidth of ~20 nm around 808 nm under full sunlight is ~60 photons nm^−2^ s^−1^ (*6*, *24*), while that of our pseudothermal light at rate 1 is ~3.3 photons nm^−2^ s^−1^ (see Materials and Methods), a factor of ~20 lower. This puts our experiments in the weak light regime where photosynthesis is constrained by the value of the incident photon flux and photosynthetic yields are proportional to this (*31*). For our heralded single photons at rate 1, we have a photon flux of ~0.93 photons nm^−2^ s^−1^. After light absorption by the B800 ring of LH2 and energy transfer processes within LH2, fluorescence emitted from the B850 ring of LH2 was collected, spectrally selected, and detected by detector 2. Each detection event in each detector was registered by a time tag with subnanosecond timing resolution, which allowed us to time, count, and correlate individual detection events of all detectors (Fig. 2B). Detailed information about the detected counts at the three *n*_p_ rates is reported in table S1.

### Fluorescence lifetime and quantum efficiency

We first investigated the effect of incident photon statistics on the lifetime and quantum efficiency of fluorescence emission from LH2, by measuring coincidence counts between herald detections at detector 1 and fluorescence detections at detector 2 as a function of their relative time delay (fig. S3). Using the herald detections as timed gates projected the incident pulses onto single photons. With a time-delay gate window equal to 1/*R*_r_ = 13.21 ns from −2 to 11.21 ns, the coincidence counts selected all the fluorescence signals within the same pulse period under conditions of incident single photons (Fig. 2, B and C). For the second mode of measurement under pseudothermal incident light, herald channel signals were used merely to represent a subset of laser pulse synchronization signals. The random coincidence counts between herald channel signals and one pulse period–delayed fluorescence (whose corresponding heralds were ignored) represented the temporal response of the fluorescence signals (Fig. 2, B and D). We note that there was a substantial amount of background in the pseudothermal incident fluorescence signal manifested by the high flat baseline in Fig. 2D, while the single-photon incident fluorescence signal had only a small portion of background (Fig. 2C) due to the gating imposed by the herald detection.

The fluorescence lifetime can then be obtained by fitting the coincidence counts in Fig. 2 (C and D, respectively) to a single exponential decay function *I*(τ) = *I*(0) × exp(−τ/τ_0_), after reconvolution with the instrument response function (Materials and Methods and tables S3 and S4). The latter is determined by the coincidence counts between heralds and incident residue selected by a different spectral filter (Materials and Methods and table S2). The experiments demonstrate that, within the error bars, the fluorescence lifetimes are identical and independent of both the photon statistics and the intensity of the incident light under our experimental conditions (Fig. 2E).

We then quantitively extracted the overall fluorescence efficiency from SPDC generation to fluorescence detection at detector 2 for both types of incident photon statistics. This efficiency encapsulates all the loss in the setup and internal dynamics in LH2 (Fig. 2F). Because we used the same setup and the same original time-tagged data, any difference in the overall fluorescence efficiency observed under different incident photon statistics must be attributed to the internal dynamics in LH2. For incident single photons, the overall fluorescence efficiency, *e*_sf_, is defined as the ratio between the heralded fluorescence photon rate and the heralded incident photon rate, i.e., *e*_sf_ = (*R*_hf_ × *P*_hf_)/[*R*_h_ × (1 + *n*_p_)], where *R*_hf_ is the heralded fluorescence channel detection rate containing background, *P*_hf_ is the weight percentage of real fluorescence signal in *R*_hf_ extracted from fittings in Fig. 2C (see Materials and Methods and table S3), *R*_h_ is the herald channel detection rate, and *R*_h_ × (1 + *n*_p_) is the corrected heralded incident photon rate taking into account that each heralded incident pulse contains 1 + *n*_p_ photons (*24*) (see section S2), where *n*_p_ is the average photon number per pulse as defined earlier. For incident pseudothermal light, the overall fluorescence efficiency, *e*_tf_, is similarly defined as the ratio between the total fluorescence photon rate and the total incident photon rate, i.e., *e*_tf_ = [*R*_hf_ × *P*_hf_ + (*R*_f_ − *R*_hf_) × *P*_f_]/(*R*_r_ × *n*_p_), where *R*_f_ is the total fluorescence channel detection rate ignoring herald detections, which again contains background, *P*_f_ is the weight percentage of real fluorescence signal extracted from the fits in Fig. 2D (see Materials and Methods and table S4), and *R*_r_ is the laser repetition rate as defined earlier. The rationale behind the expression of *e*_tf_ is that the total thermal fluorescence signal can be divided into two components. The first component is the same as that for all the heralded fluorescence photons, whose corresponding heralds (within the same pulse period) happened to be detected. The second component is the remaining signal whose corresponding heralds (within the same pulse period) were not detected. This second component has a different weight percentage, *P*_f_, in the detection rate due to the background emission, as shown in the one pulse period–delayed coincidence in Fig. 2D (also see fig. S3). Within the range of error bars, the experimentally obtained overall fluorescence efficiencies were all the same, regardless of the photon statistics and the intensity of the incident light under our experimental conditions.

### Photon statistics of the incident light and fluorescence

**Fig. 3.**
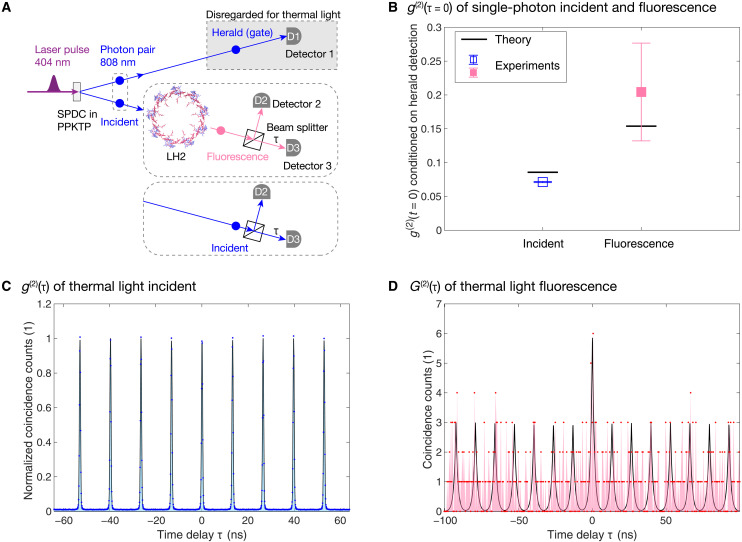
Fluorescence photon statistics are markedly different for different incident photon statistics and are essentially preserved from the incident light. (**A**) Schematics of the experiments to measure the second-order quantum coherence function, *g*^(2)^(τ) at or near τ = 0 by splitting the fluorescence (and in separate experiments the incident light) by a 50:50 beam splitter and detected by detector 2 and detector 3 in each output. (**B**) Measured (squares) and theoretically estimated (black lines, section S2) conditional *g*^(2)^(τ = 0) of the single-photon incident light and its resulting fluorescence. Error bars represent the SDs assuming Poisson statistics of the counts. (**C**) The measured *g*^(2)^(τ) near τ = 0 with 128 ps bin sizes for pseudothermal incident light. The blue dots are measured data points with 100-s integration time and the blue shadow is the area under data points. The solid black line is a fit that gives *g*^(2)^(τ = 0) = 1.0103 ± 0.0104 (Materials and Methods and table S5). (**D**) The measured unnormalized *G*^(2)^(τ) near τ = 0 with 512 ps bin sizes for fluorescence resulting from pseudothermal incident light. The red dots are measured data points with 5-h integration time, and the red shadow is the area under data points. The *G*^(2)^(τ) data are consistent with *g*^(2)^(τ) being no less than 1. However, the data are too noisy to produce a reliable fit to extract a precise value of *g*^(2)^(τ = 0). The black solid line shows the expected behavior of *g*^(2)^(τ) from ideal thermal light, with zero background counts, given the measured fluorescence lifetime (Materials and Methods).

We then investigated the effect of incident photon statistics on fluorescence photon statistics by measuring their second-order quantum coherence function *g*^(2)^(τ) at rate 1 (i.e., *n*_p_ = 0.0458). The definitions of *g*^(2)^(τ) and its unnormalized form *G*^(2)^(τ) for pulsed light are given in section S1. For large τ values (>100 ns, greater than the detector deadtime), the value of *g*^(2)^(τ) can be obtained using the standard two-detector definition (*2*, *5*) but using only detector 2, by making coincidence detections between the original detector 2 and a delayed detector 2 as a function of their relative time delay τ (see figs. S4 to S6). It is evident from these plots that *g*^(2)^(τ) = 1 at large τ for both incident and fluorescent light in all cases, neither of which show any unusual features. We note that for both forms of incident photon statistics, the measured value of *g*^(2)^(τ) is equal to 1 for fluorescence at time delays τ ~ 2 to 10 μs, implying that any contribution from the bacteriochlorophyll triplet state [lifetime ~2 to 8 μs (*32*)] to the detected fluorescence counts can be safely ignored. To measure the most interesting part of *g*^(2)^(τ) at or near zero time delay, τ = 0, the fluorescence (and, in separate experiments, the incident light) was split by a 50:50 beam splitter and detected by detectors 2 and 3 on either side (Fig. 3A).

For incident single photons (Fig. 3B), we measured the conditional *g*^(2)^(τ = 0) with standard three-detector measurements (*26*, *33*), using detector 1 as a common gate for coincidence detection between detectors 2 and 3. For this experiment, the “τ = 0” value was defined by the gate window, which is 6 ns for incident light and 10 ns for fluorescence light. For these gating timescales, the τ = 0 value is time averaged over the initial incident pulse. The conditional *g*^(2)^(τ = 0) values were obtained using the relation (*26*, *33*) *g*^(2)^(τ = 0) = (*N*_H_ × *N*_C_)/(*N*_2_ × *N*_3_), where *N*_H_ is the herald count at detector 1, *N*_C_ is the gated coincidence count between detectors 2 and 3, and *N*_2_ (*N*_3_) is the gated count at detector 2 (detector 3). The measured conditional *g*^(2)^(τ = 0) values are 0.0712 ± 0.0008 for incident light with 10-s integration time (blue square) and 0.2044 ± 0.0723 [4 SDs below the single-photon threshold of 0.5 (*5*, *26*, *33*)] for fluorescence with a 5-h integration time (red square). Both results unambiguously yield *g*^(2)^(τ = 0) < 0.5, clearly showing that both the incident light and the fluorescence were single photons (*24*). We compare these values with the predictions of simple analytical models that enable estimation of the incident and fluorescent *g*^(2)^(τ = 0) values for the heralded single-photon experiments (section S2). For the incident light, we used the simple model developed in our earlier work (*24*) that takes into account the effect of the heralding in removing the zero-photon component of the SPDC photon distribution, generating a renormalized thermal distribution and the analytic expression *g*^(2)^(τ = 0) = 2*n*_p_/(1 + *n*_p_). For rate 1, with *n*_p_ = 0.0458, this results in an estimated value *g*^(2)^(τ = 0) = 0.0857, shown as the lower black horizontal line in Fig. 3B. For the fluorescence, we developed an improved model that takes into account the small but finite background emission in heralded detection, resulting in the modified analytic expression *g*^(2)^(τ = 0) = 1 − (1 − *n*_p_^2^)/(1 + *n*_p_ + *n*_b_)^2^, where *n*_b_ is the average photon number of the background in heralded fluorescence and the background is assumed to follow Poisson statistics. Using the value *n*_b_ = 0.0402 obtained from the fitting in Fig. 2C (see table S3 caption) results in an estimated value *g*^(2)^(τ = 0) = 0.154, shown as the upper black horizontal line in Fig. 3B. These theoretical estimates are seen to agree well with the experimental values for the experiment with single incident photons.

For the case of incident pseudothermal light, i.e., ignoring detector 1 (Fig. 3, C and D), we measured the values *g*^(2)^(τ) near τ = 0 with standard two-detector measurements (*2*, *5*), by making coincidence detection between detectors 2 and 3 as a function of their relative time delay τ. The measured *g*^(2)^(τ) of the incident light (Fig. 3C) shows periodic peaks spaced by 1/*R*_r_ = 13.21 ns, due to the pulsed nature of the source. The corresponding *g*^(2)^(τ = 0) values can be obtained by calculating the ratio between the area of the center peak at τ = 0 and the average area of side peaks obtained via fitting (see Materials and Methods, section S1, and table S5). This fitting gave *g*^(2)^(τ = 0) = 1.0103 ± 0.0104, instead of the ideal value of 2 at exactly zero time delay that is expected for thermal light. This is because the pseudothermal light from our SPDC is multimode thermal light, giving rise to a *g*^(2)^(τ = 0) value between 1 and 2 (*28*, *34*). Because the photons in the two output arms of our SPDC setup are correlated in frequency, when we ignore the photons in the herald arm (i.e., tracing over the herald photon degrees of freedom), the state of the light in the incident arm becomes a mixed state of different frequency modes, with a thermal distribution of photon number for each frequency mode. Detailed analysis of the pseudothermal light generated by our SPDC setup, including dispersion effects in the optical fiber that stretch the SPDC pump laser pulse to approximately 20 ps (section S5), predicts a value *g*^(2)^(τ = 0) = 1.001, consistent with the experimentally measured value. As discussed in the Supplementary Materials, the multimode character can also be understood in the time domain as deriving from the fact that the SPDC pump laser pulse is much longer than the timescale of SPDC generation, resulting also in a large number of temporal modes. One can also rationalize the lower *g*^(2)^(τ = 0) value of multimode thermal light in both time and frequency representations using the following simplified picture. A single mode thermal light has *g*^(2)^(τ = 0) = $\frac{\left\langle n\left(n-1\right)\right\rangle }{{\left\langle n\right\rangle }^{2}}=2$ . A finite number *M* of such thermal modes, all having identical photon number distributions, will then yield a *g*^(2)^(τ = 0) value for the total photon number distribution of $\frac{\left\langle \sum_{i=1}^{M}\sum_{j=1}^{M}n_{i}\left(n_{j}-1\right)\right\rangle }{{\left\langle \sum_{i=1}^{M}n_{i}\right\rangle }^{2}}=1+\frac{1}{M}\;$ . Thus, the multimode nature tends to rapidly reduce the *g*^(2)^(τ = 0) value, from the single mode value of 2 to values approaching 1 as the number of modes *M* increases.

For the fluorescence resulting from incident pseudothermal light, we report the unnormalized second-order correlation function *G*^(2)^(τ) in Fig. 3D, because the unheralded data are too noisy to produce a reliable fit for the normalized *g*^(2)^(τ), due to the very low number of counts and the presence of a substantial amount of constant background emission in the fluorescence channel (Fig. 3D, compare with fig. S7 for the unnormalized incident correlation function). Nevertheless, these data imply a value of *g*^(2)^(τ = 0) that is not less than 1 and is consistent with thermal light, implying that the photon statistics are essentially preserved from the incident light to the fluorescence emission and are not fundamentally changed by the energy transfer processes in LH2. More detailed estimates of the value of *g*^(2)^(τ = 0) and, in particular, analysis of the excess counts in the central 2-ns region of Fig. 3D would require a reduction in the fluctuations and background emission, as discussed further below.

## DISCUSSION

Under the weak light illumination conditions and concentration of the LH2 sample in our experiments, it is a good approximation to assume that each photon is absorbed by a different LH2 complex. Therefore, it is to be expected that the fluorescence lifetime and quantum efficiency are the same, regardless of the intensity and photon statistics of the incident light. However, the experiments show that the photon statistics of the fluorescent light are not independent of the photon statistics of the incident light. Rather, the photon statistics are essentially conserved by the light-harvesting dynamics so that the fluorescent light shows the same type of photon statistics as the incident light.

Because the photon statistics describe the dynamics of the photon stream, in principle, the internal dynamics following light-matter interactions in a complex system could change the statistics of the fluorescent photons relative to the statistics of the incident light. A well-known example of the change in statistics of fluorescent photons under illumination by strong light intensity is coherent laser emission under pumping with incoherent light (*35*). In strong light, photon statistics can even influence the quantum dynamics of light-driven processes (*36*). Under less strong but still appreciable light illumination levels, fluorescence blinking phenomena observed in quantum dots and large molecules are known to alter fluorescence photon statistics on the blinking timescale (*37*–*39*), superradiant and superfluorescent effects have been seen to change the statistics of light emitted from NV centers irradiated with coherent light (*40*), and coherent excitation of single light-harvesting complexes does result in single-photon emission (*41*). However, to our knowledge, there are no previous studies at the ultralow excitation rates of the current work, with pulsed sources containing either single photons or average photon numbers considerably less than 1. In this regime, our experiments show that for both single-photon excitation and pseudothermal excitation, an ensemble of light-harvesting systems passes on the statistics of the incident light to the statistics of emitted fluorescence.

The excess counts in the *G*^(2)^(τ) values near τ = 0 on excitation by pseudothermal light (Fig. 3D), suggesting some degree of bunching, raise interesting questions as to whether some additional coherent effect is at play in this experiment. At these ultralow excitation rates with primarily 0, 1, or 2 photons per pulse, the relevant factors determining the observed fluorescent photon statistics are the number of excited emitters in the sample, the couplings between emitters, the timescale of their coherent evolution, and the strength of dephasing of their emissive states, as well as the times on which the second-order correlations are measured. In principle, even under these ultralow excitation rates, the finite numbers of emitters, collective emission effects, coherent energy transfer between emitters, and emission from multiple modes in the B850 rings can all increase the value of *g*^(2)^(τ) at exactly zero delay time τ = 0 under multimode thermal or pseudothermal excitation, even in the presence of dephasing. However, *g*^(2)^(τ) decays rapidly as |τ| increases on the timescale of dephasing of the emitter state: When it is averaged over substantially longer timescales, e.g., due to slow detector response (~350 ps in our experiments), the zero and short time delay structure in *g*^(2)^(τ) due to all of these effects is buried. The nature and dynamics of excitonic coherences in LH2 have been extensively studied, with early ultrafast measurements showing that excitonic coherence in the B850 ring of LH2 is limited by dephasing times ranging from 20 fs under ambient conditions (*42*) to a low temperature value of 6.6 ps for the red-edge band of B850 (*43*), while vibronic and electronically induced vibrational coherences are limited by vibrational dephasing times of a few picoseconds (*44*). While measurements of *g*^(2)^(τ = 0) obtained with averaging times constrained by ~350-ps time resolution cannot reflect such effects for LH2, measurements using detectors having substantially faster timescale resolution of a few picoseconds could, in principle, probe matter and photon coherences, as well as vibronic dynamics relating to energy transfer, via accurate measurements of *g*^(2)^(τ) and *g*^(2)^(τ = 0) on picosecond timescales, particularly if extended to photon statistics measurements of emitted light that are made with frequency or polarization resolution (*45*–*48*). One intriguing possibility for a larger than 1 value of *g*^(2)^(τ = 0) integrated over timescales of 0.5 ns or more is coherent coupling of the dominant *k* = −1 emitting excitonic state of the B850 ring to the local modes of the protein backbone that are responsible for the modulation of energies within the B850 ring (*49*). Such protein backbone dynamics are characterized by nanosecond timescales and are usually described by overdamped vibrations accompanied by diffusive rotation of side chains (*50*). More experiments, particularly with detectors allowing higher time resolution, are needed to better confirm and characterize the behavior of *g*^(2)^(τ) on subnanosecond timescales to probe the possibility of coherent dynamic coupling between the emitting states in the B850 ring and the LH2 protein backbone.

To summarize, using a time-resolved photon-counting QLS that can generate either single photons or pseudothermal light, we have experimentally characterized the effect of photon statistics of weak incident light on the photosynthetic light harvesting by an ensemble of LH2 complexes from the purple bacterium *R. sphaeroides* under ambient conditions, by correlating these incident photon statistics with those of the corresponding fluorescent light that is emitted after excitonic energy transfer in the LH2 complexes. After carefully analyzing the incident light, the fluorescence signals, and their correlations, we found that under ultralow excitation rates, the fluorescence lifetime and quantum efficiency were the same and independent of photon statistics, but that the fluorescence photon statistics measured by the *g*^(2)^(τ = 0) values differ for the two experiments and reflect the different incident photon statistics. For single incident photons, both fluorescent and incident *g*^(2)^(τ = 0) values are <0.5 (single photons), while for pseudothermal incident light, both the incident and fluorescent light are characterized by *g*^(2)^(τ = 0) values consistent with thermal light.

Our results indicate that ensemble average properties such as lifetime and efficiency are not affected by photon statistics of incident light under ultraweak illumination conditions and that the light-harvesting dynamics do not modify the photon statistics of the emitted fluorescence, when this is averaged over timescales larger than the emitter dephasing rates. We can expect that this will also hold for ultraweak coherent laser excitation (*51*, *52*) and ultraweak incoherent pumping. This first experiment correlating *g*^(2)^(τ = 0) measurements for fluorescent and incident light motivates further studies in the future with better time resolution to overcome the limitations due to the slow detectors and noisy fluorescence measurements in the current work. In particular, experiments with weak continuous thermal sources and detectors with ultrafast time resolution are needed for more accurate measurements of *g*^(2)^(τ)—for delay times within 350 ps to probe the possibility of coherent coupling to protein backbone modes, and within a couple of picoseconds or less to get inside the vibronic dephasing times of light-harvesting systems. We can expect that studies seeking to detect coherence within light-harvesting complexes will be best carried out on single complexes to avoid the potential effects of collective emission, but ensemble studies with higher time resolution will also be valuable, for probing collective effects at ultraweak illumination as well as energy transfer between complexes and multistate emission. Overall, the current experimental study demonstrates the potential of QLS to harness photon statistics to gain insights into the molecular quantum dynamics of a wide range of systems including photosynthetic light-harvesting systems and to answer questions relevant to the microscopic mechanisms of light-initiated processes in biological systems.

## MATERIALS AND METHODS

### LH2 sample

LH2 was purified from wild-type *R. sphaeroides* ATCC2.4.1 cells provided by the Blankenship Lab at Washington University in St. Louis, as previously described (*53*, *54*). Purified LH2 complexes were solubilized in 20 mM tris-HCl and 0.1% n-Dodecyl β-D-maltoside (DDM) (Anatrace), pH 7.5 buffer, concentrated to 31.4 μM, and stored at −80°C in 500-μl aliquots until used for experiments.

### The experimental setup

Our time-resolved photon-counting QLS setup has been described in detail elsewhere (*24*). Here, we provide a summary. The setup consists of three major optical modules—an SHG (second harmonic generation) module to prepare the pump laser, an SPDC module to generate the squeezed vacuum state, and a fluorescent module for fluorescence excitation and collection—as well as detection electronics. A femtosecond laser with repetition rate *R*_r_ = 75.7 MHz (Mira 900, Coherent) at 808 nm is first converted up to 404 nm via a barium borate (BBO) crystal in the SHG module. The spatially and spectrally filtered 404-nm laser is then directed via a single mode fiber to the SPDC module to produce squeezed vacuum state at 808 nm, through type II phase matching in a 30-mm-long periodically poled potassium titanyl phosphate (PPKTP) crystal (poling period, 9.825 μm) (Raicol Crystals Ltd). The two arms of the squeezed vacuum state have orthogonal polarizations and are spatially separated by a polarizing beam splitter and collected into single mode fibers. The herald arm is detected by detector 1 (SPCM AQRH 13, Excelitas). The incident arm is directed to the fluorescence module and focused through a microscope objective (60×, numerical aperture = 0.70) to excite the LH2 sample, which contains about 7 × 10^4^ LH2 complexes in solution within an interaction volume of ∼3.6 μm^3^ at room temperature under ambient conditions [see Supplementary Materials of (*24*)]. The incident light focus area is a disk with estimated area 1.0645 μm^2^, resulting in an average photon flux for rate 1 (0.0458 photons per pulse) of 0.0458 × 75.7/1.046 ~ 3.3 photons nm^−2^ s^−1^. Both incident residue and fluorescence are collected by the same objective. Then, either incident residue or fluorescence is spectrally selected by a filter that blocks the other, and detected by detector 2 (SPCM AQRH 16, Excelitas). Each detection event from the two detectors is recorded with ~350 ps resolution by a time tagger (Time Tagger Ultra, Performance Edition, Swabian Instruments) with channel dead time setting 86 ns. For measuring the second-order quantum coherence function *g*^(2)^(τ) at or near τ = 0, the incident or fluorescent light is split by a 50:50 fiber beam splitter and connected to detector 2 (SPCM AQRH 16, Excelitas) and detector 3 (SPCM AQRH 16, Excelitas) on each output. As above, each detection event from the three detectors is recorded with ~350 ps resolution by a time tagger (Time Tagger Ultra, Performance Edition, Swabian Instruments) with channel dead time setting 86 ns.

### Data analysis

#### Lifetime and efficiency

The fluorescence decays in Fig. 2 (C and D) were fitted by exponentially modified Gaussian distributions (that is, convolution between single exponential and Gaussian functions) with four free parameters: the overall height, the peak position, the decay lifetime, and a constant background, which directly produced the lifetime values. The Gaussian width parameter in the above fitting came from fitting the instrument response function by a Gaussian distribution. The weight percentages of fluorescence signal, *P*_hf_ and *P*_f_, were obtained by the ratio between background-subtracted fluorescence count and total fluorescence count for single-photon incident data in Fig. 2C and pseudothermal light incident data in Fig. 2D, respectively. See tables S2 to S4 for more details of the fittings, including the obtained parameters.

#### Second-order quantum coherence function g^(2)^(τ) at or near τ = 0

The raw coincidence count data were fit by nine equally spaced Gaussian peaks with six free parameters: the time interval between adjacent peaks, the position of the center peak, the height of the center peak, the equal height of all side peaks, the equal width of all peaks, and a constant background. The *g*^(2)^(τ) data shown in Fig. 3C were normalized by the resulting equal height parameter of all side peaks. See table S5 for explicit details of the fits, including the parameters obtained from the fit. The idealized *G*^(2)^(τ) curve in Fig. 3D shows 15 equally spaced, double-sided single-exponential peaks with no background, each having the decay constant from the fluorescence lifetime in Fig. 2E. The center peak is scaled to six and is twice as high as a side peak.

## Supplementary Materials

### This PDF file includes:

Supplementary Text Figs. S1 to S11 Tables S1 to S5 References
