# Supplementary material for: Comparing photosynthetic light harvesting of single photons and pseudothermal light under ultraweak illumination
Source: Sci Adv. 2025 Nov 14;11(46):eadz2616. doi: 10.1126/sciadv.adz2616 (PMC12617509; doi:10.1126/sciadv.adz2616)
Supplement: Supplementary file 1 — Supplementary Text Figs. S1 to S11 Tables S1 to S5 References [file sciadv.adz2616_sm.pdf]

Science Advances
AAAS

Supplementary Materials for

Comparing photosynthetic light harvesting of single photons and pseudothermal light under ultraweak illumination

Quanwei Li *et al.*

Corresponding author: K. Birgitta Whaley, whaley@berkeley.edu; Graham R. Fleming, grfleming@lbl.gov

Sci. Adv. **11**, eadz2616 (2025)
DOI: 10.1126/sciadv.adz2616

This PDF file includes:

Supplementary Text
Figs. S1 to S11
Tables S1 to S5
References

1 Definitions of $g^{(2)}$ and $G^{(2)}$ for general and pulsed light sources

The second-order quantum coherence function $g^{(2)}$ is a function of two time variables t_1 and t_2, defined as (*4*).

$$g^{(2)}(t_1,t_2) = \frac{\langle a^\dagger(t_1)a^\dagger(t_2)a(t_2)a(t_1)\rangle}{\langle a^\dagger(t_1)a(t_1)\rangle\langle a^\dagger(t_2)a(t_2)\rangle}, \tag{1}$$

where $a(t)$ is the photon annihilation operator at retarded time t. The unnormalized second-order correlation function $G^{(2)}$ is defined as

$$G^{(2)}(t_1,t_2) = \langle a^\dagger(t_1)a^\dagger(t_2)a(t_2)a(t_1)\rangle. \tag{2}$$

$G^{(2)}(t_1,t_2)$ can be interpreted as the joint intensity at times t_1 and t_2. For stationary light, we define the single time variable $G^{(2)}(\tau)$ as $G^{(2)}(t_1,t_1+\tau)$. For a general state of light that is not necessarily stationary, one can still measure or compute the probability that two photons are detected at time τ apart from one another. Within some detection time interval T_d, the probability that two photons are detected at time τ apart from one another is proportional to the integral

$$F(\tau) = \int_0^{T_d} dt_1\, G^{(2)}(t_1,t_1+\tau). \tag{3}$$

The output light of our SPDC setup and the fluorescent light driven by the periodically pulsed light excitation are both periodically pulsed, with period T.

Properties of periodically pulsed light are invariant under time translation of νT, for ν integer. Therefore $g^{(2)}(t_1,t_2) = g^{(2)}(t_1+\nu T, t_2+\nu T)$, $G^{(2)}(t_1,t_2) = G^{(2)}(t_1+\nu T, t_2+\nu T)$, and $F(\tau)$ will show a periodic sequence of peaks centered at $\tau = \nu T, \nu = 0, 1, ...$ If photon counting is performed for a long time (i.e., $T_d \to \infty$), then in principle $F(\tau)$ becomes

$$F(\tau) = \sum_{\nu=0}^{\infty}\int_0^T ds\, G^{(2)}(\nu T+s, \nu T+s+\tau) = \sum_{\nu=0}^{\infty}\int_0^T ds\, G^{(2)}(s,s+\tau), \tag{4}$$

from which we can conclude the proportionality relationship

$$F(\tau) \propto \int_0^T ds\, G^{(2)}(s,s+\tau), \tag{5}$$

allowing $g^2(\tau)$ to be measured from individual pulses. In our experiments, the pulses are short in time (~ 20 ps) compared to the repetition period T (~ 13 ns), so photon detection events only occur near where the pulses are, and there is no photon detection event far away from the pulses. The pulses are centered at $(\nu+\frac{1}{2})T$. $G^{(2)}(t_1,t_2)$ is then concentrated at points near $(t_1,t_2) = ((\mu+\frac{1}{2})T, (\nu+\frac{1}{2})T)$, where both μ and ν are integers, (see Figure S1(a)). Far away from the points $(t_1,t_2) = ((\mu+\frac{1}{2})T, (\nu+\frac{1}{2})T)$, $G^{(2)}(t_1,t_2)$ is close to zero, since no photon detection event occurs far away from the pulses. Lines of constant τ appear as diagonal lines in Figure S1(a). We can see that $F(\tau)$, the integral of $G^{(2)}(t_1,t_1+\tau)$, has significant amplitude only at τ values near integer multiples of T. An example of $F(\tau)$ is shown in Figure S1(b) below.

To distinguish from the common definitions of $g^{(2)}(\tau) = g^{(2)}(t_1,t_1+\tau)$ and $G^{(2)}(\tau) = G^{(2)}(t_1,t_1+\tau)$, which apply strictly only to stationary light, we denote the normalized second-order quantum coherence function for periodically pulsed light as $g_p^{(2)}(\tau = \nu T)$. The subscript p emphasizes that the light source is periodically pulsed and the time difference τ is set to be an integer multiple of the period T. (We will use the notation $g_p^{(2)}(\tau)$ only in this section and Secs. 4 - 5 of the Supplementary Materials below, to be precise in the mathematical derivation. In the main manuscript and remaining parts of the Supplementary Materials we leave out the subscript p for simplicity.) Then $g_p^{(2)}(\tau = \nu T)$ is defined as the ratio

$$g_p^{(2)}(\tau = \nu T) = \frac{\int_{(\nu-1/2)T}^{(\nu+1/2)T} d\tau\, F(\tau)}{\lim\limits_{\mu\to\infty}\displaystyle\int_{(\mu-1/2)T}^{(\mu+1/2)T} d\tau'\, F(\tau')}, \tag{6}$$

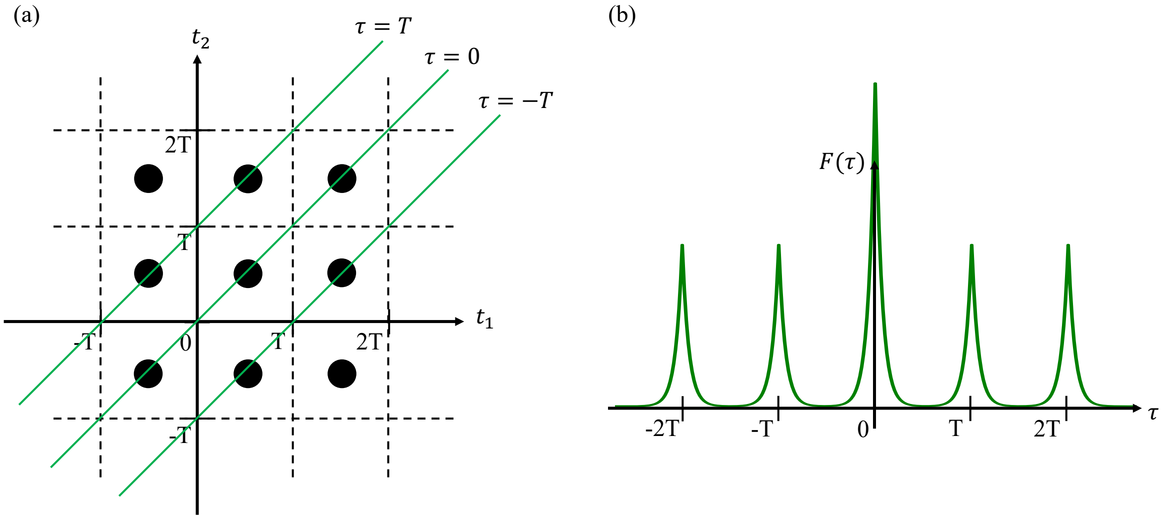

Figure S1: (a) Schematics of $G^{(2)}(t_1, t_2)$ for pulsed light with periodicity T. If the durations of the light pulses are much smaller than the pulse repetition period T, then $G^{(2)}(t_1, t_2)$ is nonzero only around the points $(t_1, t_2) = ((\mu + \frac{1}{2})T, (\nu + \frac{1}{2})T)$, where ν and μ are integers. Black dots represent regions where $G^{(2)}(t_1, t_2)$ is nonzero. Green lines represent lines of constant $\tau = t_2 - t_1$. (b) An example of $F(\tau)$ in the case of periodically pulsed light. If one measures the coincidence counts only as a function of the time difference τ between photon detection events, then the coincidence count probability $F(\tau)$ is obtained by integrating over the first time variable in $G^{(2)}(t_1, t_2)$ (see Eq. (3)).

where the numerator is the integral of the $\tau = \nu T$ peak of $F(\tau)$, and the normalization factor in the denominator is the integral of a peak at large enough time difference $\tau' = \lim_{\mu\to\infty} \mu T$ so that two-photon detection events in the denominator are independent, allowing this to be factorized.

We are primarily interested in $g_p^{(2)}(\tau = 0)$ (i.e., the case of $\nu = 0$) because in our experiment photons in different pulses are independent (apart from the pulse periodicity) and only the $\tau = 0$ peak (i.e., when two photons are in the same pulse) exhibits non-trivial second-order quantum coherence. So $g_p^{(2)}(\tau = 0)$ can then be calculated from the area of the central peak at $\tau = 0$, ie., $F(\tau)$, divided by the area of a distant side peak at large μ. Quantitatively, using Eq. (5), the numerator of Eq. (6) becomes

$$\int_{-T/2}^{T/2} d\tau\, F(\tau) \propto \int_{-T/2}^{T/2} d\tau \int_0^T dt_1\, G^{(2)}(t_1, t_1 + \tau), \tag{7}$$

i.e., the number of coincidences in the $\nu = 0$ pulse. The region of integration in Eq. (7) is shown in Figure S2. Since the integral is dominated by (t_1, t_2) close to $(T/2, T/2)$, we can approximate this integral by another integral with a rectangular integration region, i.e.,

$$\begin{aligned} \int_0^T dt_1 \int_0^T dt_2\, G^{(2)}(t_1, t_2) &= \int_0^T dt_1 \int_0^T dt_2\, \langle a^\dagger(t_1) a^\dagger(t_2) a(t_2) a(t_1)\rangle \\ &= \langle n(n-1)\rangle, \end{aligned} \tag{8}$$

where n is the photon number in the time interval $[0, T]$. We note that in our $g_p^{(2)}(\tau = 0)$ measurements for heralded incident and fluorescent light, we have chosen the gating windows T_g to be slightly shorter than the pulse period T. Specifically, T_g is 6 ns for incident light and 10 ns for fluorescent light. This corresponds to replacing the integration bounds $[0, T]$ in Eq. (8) with $[T/2 - T_g/2, T/2 + T_g/2]$, i.e., an interval with width T_g centered on $T/2$. Since the pulse durations are still much shorter than T_g, this replacement does not change the value of Eq. (8).

Similarly, the integral of the $F(\tau)$ peak centered at μT is taken as

$$\int_{\mu T - T/2}^{\mu T + T/2} d\tau \, F(\tau). \tag{9}$$

Similar to the derivation of Eq. (8), this integral can be shown for μ large enough that the photons in the two pulses at $\nu = 0$ and at $\nu = \mu$ are independent, to be proportional to

$$\begin{aligned} \int_0^T dt_1 \int_{\mu T}^{(\mu+1)T} dt_2 \, G^{(2)}(t_1, t_2) &= \int_0^T dt_1 \int_{\mu T}^{(\mu+1)T} dt_2 \, \langle a^\dagger(t_1) a(t_1) \rangle \langle a^\dagger(t_2) a(t_2) \rangle \\ &= \langle n \rangle^2, \end{aligned} \tag{10}$$

Combining Eqs. (8) and (10), yields

$$g_p^{(2)}(\tau = 0) = \frac{\langle n(n-1) \rangle}{\langle n \rangle^2} = \frac{\langle n^2 \rangle - \langle n \rangle}{\langle n \rangle^2}, \tag{11}$$

taking the same form as that of $g^{(2)}(0)$ in single-mode quantum optics. However, here n denotes the number of photons in a pulse, rather than the intensity of a continuous wave, and the pulses are derived from a continuum of frequency modes, described by multi-mode quantum optics with associated mode functions (*4*).

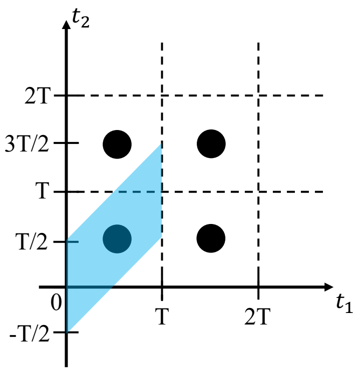

Figure S2. Integration region in Eq. (7)**.**

2 Theoretical estimation of $g^{(2)}(\tau = 0)$ for heralded incident and fluorescent light

To obtain the second-order quantum coherence function at zero time delay, $g^{(2)}(\tau = 0)$, Eq. (11) shows that we need the average values of n and n^2 in each pulse, which requires knowledge of the distribution of the photon number n.

2.1 Heralded incident photons

This subsection describes the same simple model developed in our previous work (*24*), which is provided here for convenience and completeness.

The output of a type II spontaneous parametric down conversion (SPDC) is a degenerate two-mode squeezed vacuum state (*4*). Tracing out one of the modes leads to a thermal photon number distribution for the other mode, i.e., in a single arm of the SPDC, that is given by

$$P(n_p, n) = \frac{n_p^n}{(1+n_p)^{n+1}}. \tag{12}$$

Here $n_p > 0$ is the average number of photon pairs per pump pulse (which is equal to the average photon number in one of the two arms, per pump pulse), and $n = 0, 1, 2, 3\ldots$ is the number of photons in the state. As noted in the main text, in the context of these experiments on light harvesting systems, we refer to Eq. (12) as a pseudothermal distribution, to acknowledge its origin from a non-linear source rather than sunlight.

The herald detection then collapses the squeezed vacuum state, removes the zero-photon component of $P(n_p, n)$, and projects the incident beam onto the following renormalized pseudothermal distribution:

$$P_h(n_p, n) = 0 \qquad \text{for } n = 0, \tag{13}$$

$$P_h(n_p, n) = \frac{(1+n_p)}{n_p}\frac{n_p^n}{(1+n_p)^{n+1}} \qquad \text{for } n = 1, 2, 3, \ldots \tag{14}$$

With this projection the heralded incident beam has average values $\langle n \rangle$ and $\langle n^2 \rangle$ as follows:

$$\langle n \rangle = \sum_{n=1}^{+\infty} n \times P_h(n_p, n) = 1 + n_p \tag{15}$$

$$\langle n^2 \rangle = \sum_{n=1}^{+\infty} n^2 \times P_h(n_p, n) = 1 + 3n_p + 2n_p^2. \tag{16}$$

Inserting $\langle n \rangle$ and $\langle n^2 \rangle$ into the general expression for $g^{(2)}(\tau = 0)$, we obtain the following expression:

$$g^{(2)}(\tau = 0) = \frac{2n_p}{1+n_p}, \tag{17}$$

without any free parameters. For the experiment employing Rate 1 with average photon number per pulse $n_p = 0.0458$, we obtain the value $g^{(2)}(\tau = 0) = 0.0857$ that is reported in Figure 3B of the main text.

2.2 Heralded fluorescent photons

The heralded fluorescent photons additionally contain a small but noticeable amount of background emission (Figure 2C of the main text), which is assumed to follow a Poisson distribution with average photon number n_b, i.e.,

$$P_b(n_b, n) = e^{-n_b}\frac{n_b^n}{n!}. \tag{18}$$

Now we consider that the heralded fluorescent photons have two contributions, namely, the real fluorescent signal with a photon number n_1 following the renormalized pseudothermal distribution $P_h(n_p, n_1)$ that was derived in Section 2.1 above, and a background emission with photon number n_2 following the Poisson distribution $P_b(n_b, n_2)$. Then we obtain

$$\langle n \rangle = \sum_{n_1=1}^{+\infty}\sum_{n_2=0}^{+\infty}(n_1+n_2)P_h(n_p, n_1)P_b(n_b, n_2) = 1 + n_p + n_b \tag{19}$$

$$\langle n^2 \rangle = \sum_{n_1=1}^{+\infty}\sum_{n_2=0}^{+\infty}(n_1+n_2)^2P_h(n_p, n_1)P_b(n_b, n_2) = 1 + 3n_p + 3n_b + 2n_pn_b + 2n_p^2 + n_b^2. \tag{20}$$

Inserting $\langle n \rangle$ and $\langle n^2 \rangle$ into the general expression for $g^{(2)}(\tau = 0)$, we obtain the following expression:

$$g^{(2)}(\tau = 0) = 1 - \frac{1 - n_p^2}{(1 + n_p + n_b)^2}. \tag{21}$$

For the experiment employing Rate 1 we have average photon number $n_p = 0.0458$, and from the fits to Figure 2C in the main text, we extract the value $n_b = 0.0402$ for the average photon number of the background (see the caption of Table S3). Using these values $n_p = 0.0458$ and $n_b = 0.0402$ in Eq. (21) yields the value $g^{(2)}(\tau = 0) = 0.154$ that is reported in Figure 3B.

3 Experimental Details

Incident rates	Rate 1 ($n_p = 0.0458$)	Rate 2 ($n_p = 0.0214$)	Rate 3 ($n_p = 0.0103$)
Measured herald rate R_h (counts/s) at Detector 1	9.74×10^5	4.74×10^5	2.32×10^5
Measured dark count rate D_h (counts/s) at Detector 1 (herald channel)	471	471	471
Calculated ratio D_h/R_h	0.0484%	0.0994%	0.203%
Measured total fluorescence rate R_f (counts/s) at Detector 2	788	374	199
Measured heralded fluorescence rate R_{hf} with gate window 13.21 ns from -2 to 11.21 ns (counts/s)	122	57.9	27.9
Measured dark count rate D_{f2} (counts/s) at Detector 2 (fluorescence channel)	30	30	30
Calculated ratio D_{f2}/R_f	3.81%	8.02%	15.1%
Calculated contribution from dark counts to total heralded fluorescent counts with 13.21 ns gate window: $D_{f2} \times R_h \times [13.21\,\mathrm{ns}]/R_{hf}$	0.315%	0.325%	0.328%

Table S1. Summary of experimentally measured count rates for the data in Figure 2 of the main text.

The detector dark counts follow Poisson distributions and the measured dark count rates shown here are long-time averages of these. The contribution from dark counts in the herald counts is found to be less than 0.3 % and can be safely ignored. The ratio of dark counts to total fluorescent counts is noticeable and is listed here for convenience, but this ratio is largely irrelevant for the coincidence counts, which are carefully analyzed separately. Because of the gated nature of the heralded fluorescence photon detection, the contribution from dark counts is found to be less than 0.4%, which is negligible.

Parameters	Values (with 95% confidence bounds)
Peak height (1) a_1	1 (0.9905, 1.01)
Peak position (ns) b_1	-0.01389 (-0.01673, -0.01104)
Peak width (ns) c_1	0.3512 (0.3472, 0.3552)
**Goodness of Fitting**	**Values**
sse	0.0057
rsquare	0.9982
dfe	100
adjrsquare	0.9982
rmse	0.0076

Table S2. Fitting parameters obtained for the instrument response function shown as the black dashed line in Figure 2C of the main text.

The fitting model is a Gaussian peak with 3 free parameters:

$$F_0(x) = a_1 e^{-((x-b_1)/c_1)^2}. \tag{22}$$

The fitted peak width value $c_1 = 0.3512$ is used for further fitting of the data in Figure 2C and Figure 2D of the main text, as reported in detail in Table S3 and Table S4.

Parameters	Values (with 95% confidence bounds) at Rate 1 ($n_p = 0.0458$)	Values (with 95% confidence bounds) at Rate 2 ($n_p = 0.0214$)	Values (with 95% confidence bounds) at Rate 3 ($n_p = 0.0103$)
Overall height (1), A	1.48253 (1.468102, 1.496958)	1.482514 (1.466649, 1.498378)	1.448029 (1.429204, 1.466855)
Peak position (ns), b_1	-0.104781 (-0.110256, -0.099307)	-0.058841 (-0.064875, -0.052806)	-0.043614 (-0.05092, -0.036308)
Constant background (1), d_1	0.007412 (0.005154, 0.009669)	0.003479 (0.000983, 0.005974)	0.002306 (-0.000633, 0.005246)
Decay lifetime (ns), τ_0	1.29831 (1.277964, 1.318656)	1.304237 (1.281736, 1.326737)	1.295927 (1.268812, 1.323043)
Goodness of fitting	Values at Rate 1 ($n_p = 0.0458$)	Values at Rate 2 ($n_p = 0.0214$)	Values at Rate 3 ($n_p = 0.0103$)
sse	0.007119	0.00867	0.012084
rsquare	0.99897	0.998751	0.998165
dfe	99	99	99
adjrsquare	0.998938	0.998713	0.998109
rmse	0.00848	0.009358	0.011048
Derived Parameter			
Overall fluorescence efficiency (1), e_{sf}	1.144×10^{-4}	1.167×10^{-4}	1.175×10^{-4}
confidence bounds on e_{sf}	$(1.127, 1.162) \times 10^{-4}$	$(1.147, 1.187) \times 10^{-4}$	$(1.151, 1.199) \times 10^{-4}$

Table S3. Fitting parameters obtained for the data in Figure 2C of the main text.

The fitting model is an exponentially modified Gaussian distribution, i.e., a convolution between single exponential and a Gaussian function, with 4 free parameters:

$$F_1(x) = (A/2)e^{0.3512^2/(4\tau_0^2)} \times e^{-(x-b_1)/\tau_0} \times (1 + erf((x - b_1 - 0.3512^2/(2\tau_0))/0.3512)) + d_1. \quad (23)$$

The fittings directly produce the measured fluorescence lifetime values τ_0. The Gaussian width parameter of 0.3512 in the fitting function Eq. (23) comes from fitting the instrument response function by a Gaussian distribution as reported in the caption to Table S1.

For incident single photons at each of the three rates (measured by n_p), the overall fluorescence efficiency, e_{sf}, is defined as the ratio between the heralded fluorescence photon rate and the heralded incident photon rate, i.e.,

$$e_{sf} = (R_{hf} \times P_{hf})/[R_h(1 + n_p)]. \quad (24)$$

where R_{hf} is the heralded fluorescence channel detection rate containing background (values reported in Table S1), P_{hf} is the weight percentage of real fluorescence signal in R_{hf} extracted from fittings in Figure 2C according to $P_{hf} = 1–d_1 \times$ [number of data points]/[sum of data values], R_h is the herald channel detection

rate (values reported in Table S1), and $R_h(1+n_p)$ is the corrected heralded incident photon rate taking into account that each heralded incident pulse contains $1+n_p$ photons (see Supplementary Materials Section 2), where n_p is the average photon number per pulse.

Similarly, the average photon number of the background (assumed to follow Poisson statistics) in heralded fluorescence, n_b, can be obtained from solving the following equation:

$$n_b/(1+n_p+n_b) = d_1 \times [\text{number of data points}]/[\text{sum of data values}]. \tag{25}$$

For the data at Rate 1 ($n_p = 0.0458$), the solution gives $n_b = 0.0531$ for the 13.21 ns gate window and hence $n_b = 0.0402 (= 0.0531 * 10/13.21)$ for a 10 ns gate window. The $n_b = 0.0402$ value is then used in Supplementary Materials Section 2.2 to calculate the theoretical estimation of $g^{(2)}(t = 0)$ for heralded fluorescence.

Parameters	Values (with 95% confidence bounds) at Rate 1 ($n_p = 0.0458$)	Values (with 95% confidence bounds) at Rate 2 ($n_p = 0.0214$)	Values (with 95% confidence bounds) at Rate 3 ($n_p = 0.0103$)
Overall height (1), A	1.20858 (1.177681, 1.239478)	1.217727 (1.147556, 1.287898)	1.061695 (0.93948, 1.18391)
Peak position (ns), b_1	-0.128186 (-0.142433, -0.113939)	-0.102159 (-0.134377, -0.069941)	-0.08277 (-0.151674, -0.013867)
Constant background (1), d_1	0.149849 (0.145073, 0.154624)	0.152858 (0.141937, 0.163779)	0.187808 (0.165836, 0.209781)
Decay lifetime (ns), τ_0	1.274697 (1.222343, 1.327051)	1.282391 (1.163492, 1.401291)	1.450202 (1.172085, 1.728319)
**Goodness of fitting**	**Values at Rate 1 ($n_p = 0.0458$)**	**Values at Rate 2 ($n_p = 0.0214$)**	**Values at Rate 3 ($n_p = 0.0103$)**
sse	0.031358	0.163311	0.601887
rsquare	0.993072	0.965635	0.866303
dfe	98	98	98
adjrsquare	0.99286	0.964583	0.862211
rmse	0.017888	0.040822	0.078369
**Derived Parameter**			
Overall fluorescence efficiency (1), e_{tf}	1.182×10^{-4}	1.207×10^{-4}	1.200×10^{-4}
confidence bounds on e_{tf}	$(1.142, 1.221) \times 10^{-4}$	$(1.123, 1.291) \times 10^{-4}$	$(1.035, 1.365) \times 10^{-4}$

Table S4. Fitting parameters obtained for the data in Figure 2D of the main text.

The fitting model is the same as in Table S3, an exponentially modified Gaussian distribution, i.e., a convolution between a single exponential and a Gaussian function) with 4 free parameters:

$$F_1(x) = (A/2)e^{0.3512^2/(4\tau_0^2)} \times e^{-(x-b_1)/\tau_0} \times (1 + erf((x - b_1 - 0.3512^2/(2\tau_0))/0.3512)) + d_1. \quad (26)$$

The fittings directly produce the measured lifetime values τ_0. The Gaussian width parameter of 0.3512 in the fitting function Eq. (26) comes from fitting the instrument response function by a Gaussian distribution as reported in the caption to Table S1.

For incident pseudothermal light at each incident photon Rate, the overall fluorescence efficiency, e_{tf}, is defined as the ratio between the total fluorescence photon rate and the total incident photon rate, i.e.,

$$e_{tf} = [R_{hf}P_{hf} + (R_f – R_{hf})P_f]/(R_r n_p), \quad (27)$$

where R_f is the total fluorescence channel detection rate ignoring herald detections (values reported in Table S1), which again contains background, P_f is the weight percentage of real fluorescence signal extracted from the fits in Figure 2D, according to

$$P_f = 1 – d_1 \times [\text{number of data points}]/[\text{sum of data values}], \quad (28)$$

and $R_r = 75.7$ MHz is the laser repetition rate. The rationale behind the expression for e_{tf} is that the total fluorescence signal following excitation by pseudothermal light can be divided into two components. The first component is the same as all the heralded fluorescence photons whose corresponding heralds (within the same pulse period) happened to be detected. The second component is the remaining signal whose corresponding heralds (within the same pulse period) were not detected. This second component has a different weight percentage, P_f, in the detection rate due to the background emission, as shown in the one-pulse-period-delayed coincidence in Figure 2D of the main text and in Figure S3.

Parameters	Values (with 95% confidence bounds)
Height of center peak (1) a	0.9833 (0.9762, 0.9904)
Inverse of width of all peaks (1/ns) b	8.935 (8.874, 8.996)
Constant background (1) c	0.01196 (0.01155, 0.01238)
Height of all side peaks (1) d	0.9733 (0.9703, 0.9763)
Distance between adjacent peaks (ns) e	13.21 (13.21, 13.21)
Position of center peak (ns) f	-0.06671 (-0.06751, -0.06592)
**Goodness of Fitting**	**Values**
sse	0.040490878270776
rsquare	0.998465213920078
dfe	994
adjrsquare	0.998457493668167
rmse	0.006382420388132

Table S5. Fitting parameters obtained for the data in Figure 3C of the main text.

The fitting model is 9 equally spaced Gaussian peaks with 6 free parameters, namely:

$$\begin{aligned} F_2(x) =& ae^{-b(x+f)^2} + de^{-b(x+f-e)^2} + +de^{-b(x+f+e)^2} + de^{-b(x+f-2e)^2} + de^{-b(x+f+2e)^2} \\ &+ de^{-b(x+f-3e)^2} + de^{-b(x+f+3e)^2} + de^{-b(x+f-4e)^2} + de^{-b(x+f+4e)^2} + c. \end{aligned} \tag{29}$$

Then we obtain the value for $g^{(2)}(\tau = 0)$ as the ratio of the central peak area to the area of a side peak, giving $g^{(2)}(\tau = 0) = a/d = 1.0103 \pm 0.0104$.

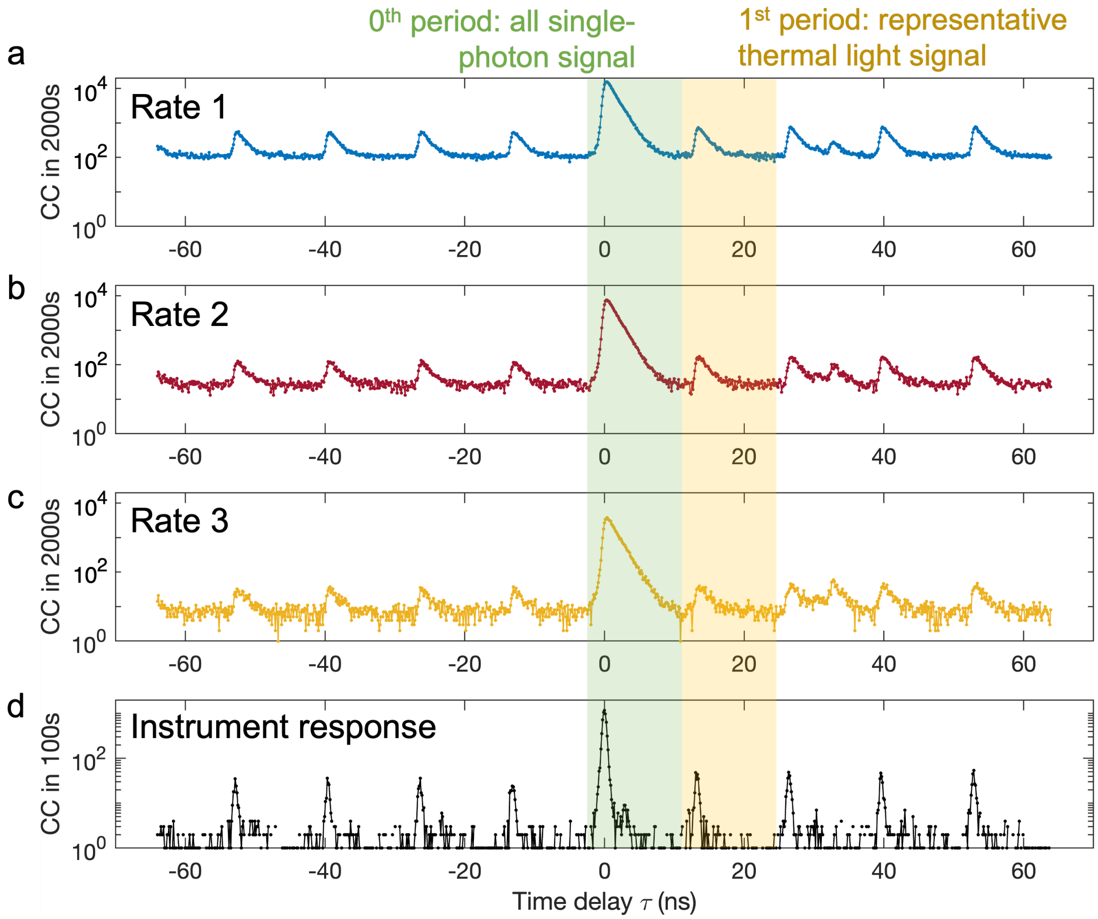

Figure S3. Source data for Figure 2.

Panels a - c: cross-correlation coincidence counts between herald detections at Detector 1 and fluorescence detections at Detector 2 as a function of their relative time delay, for the three incident rates 1 - 3, respectively, obtained with a 2000-s integration time and 128 ps bin size. Panel d: the instrument response function, represented by the cross-correlation coincidence counts between herald detections at Detector 1 and incident residue detection at Detector 2 selected by a different spectral filter as function of their relative time delay, for Rate 1 excitation with a 100-s integration time and 128 ps bin size. CC denotes coincidence counts. Repeated peaks are spaced by the laser pulse period $1/Rr = 13.21$ ns. The 0th period highlighted by green shading from -2 ns to 11.21 ns is shown in Figure 2C in the main text, which contains all the coincidence signals obtained from incident single photons. The one-pulse-period-delayed 1st period highlighted by gold shading from 11.21 ns to (11.21+13.21) ns is shown in Figure 2D in the main text, which presents representative coincidence signals obtained from incident pseudothermal light. The extra peak around 32 ns time delay is a technical artifact, likely resulting from light reflected by a surface(s) or interface(s) in the setup.

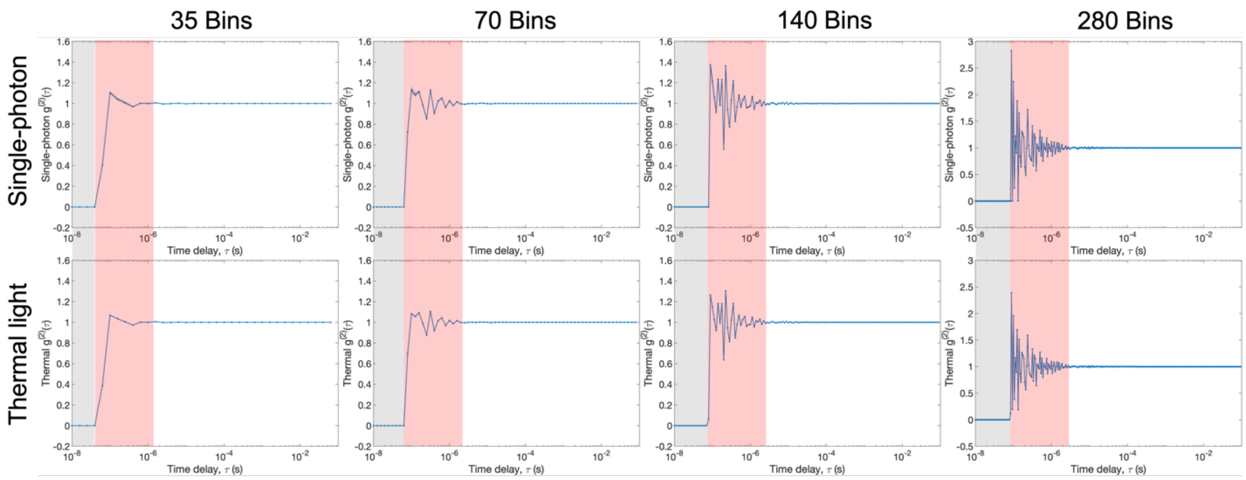

Figure S4. Second-order quantum coherence function $g^{(2)}(\tau)$ of the incident light detected by Detector 2 as a function of time delay spanning from 10 ns to 0.1 s with different total bin numbers (i.e., time resolution) at Rate 1.

The second-order quantum coherence function $g^{(2)}(\tau)$ is obtained using the standard two-detector definition but employing only Detector 2, by making coincidence detections between the original Detector 2 and a delayed Detector 2 as a function of their relative time delay τ. The delayed Detector 2 is introduced by the software of the Time Tagger module; this introduces a virtually delayed channel that is treated in the same way as a real detector channel and can be used for coincidence measurement. Blue dots and solid lines are the measured data averaged by 10 repeated data recordings, each made with a 10-s integration time. The blue shading sandwiching the data points and connecting lines marks the standard deviation of the 10 repeats, which is similar to the size of the points and is thus difficult to see. The gray shading marks the region where $g^{(2)}(\tau) = 0$ due to the 86 ns deadtime of the detector (*24*). The red shading marks the region where $g^{(2)}(\tau)$ may contain oscillating artifacts due to the periodic nature of the signals, evidenced by its dependence on the time resolutions. Overall, the data suggests that $g^{(2)}(\tau) = 1$ across the accessible time scales for both incident single photons and pseudothermal light.

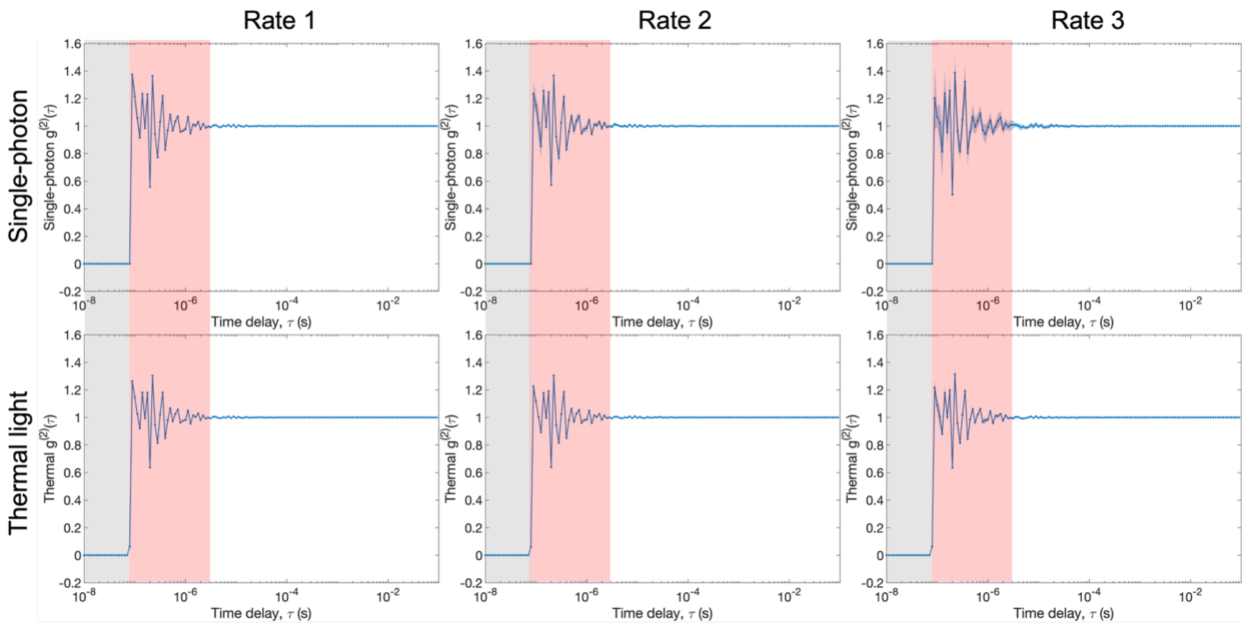

Figure S5. Second-order quantum coherence function $g^{(2)}(\tau)$, of the incident light detected by Detector 2 as a function of time delay spanning from 10 ns to 0.1 s in 140 time bins at Rate 1 to 3.

The second-order quantum coherence function $g^{(2)}(\tau)$ is obtained using the standard two-detector definition but employing only Detector 2, by making coincidence detections between the original Detector 2 and a delayed Detector 2 as a function of their relative time delay τ. The delayed Detector 2 is introduced by the software of the Time Tagger module; this introduces a virtually delayed channel that is treated in the same way as a real detector channel and can be used for coincidence measurement. Blue dots and solid lines are the measured data averaged by 10 repeated data recordings, each made with a 10-s integration time. The blue shading sandwiching the data points and connecting lines marks the standard deviation of the 10 repeats, which is similar to the size of the points and is thus difficult to see. The gray shaded region marks where $g^{(2)}(\tau) = 0$ due to the 86 ns deadtime of the detector (*24*). The red shaded region marks the region where $g^{(2)}(\tau)$ shows oscillating artifacts due to the periodic nature of the signals (see Figure S4). Overall, the data suggest that $g^{(2)}(\tau) = 1$ across the accessible time scales and incident rates for both incident single photons and pseudothermal light.

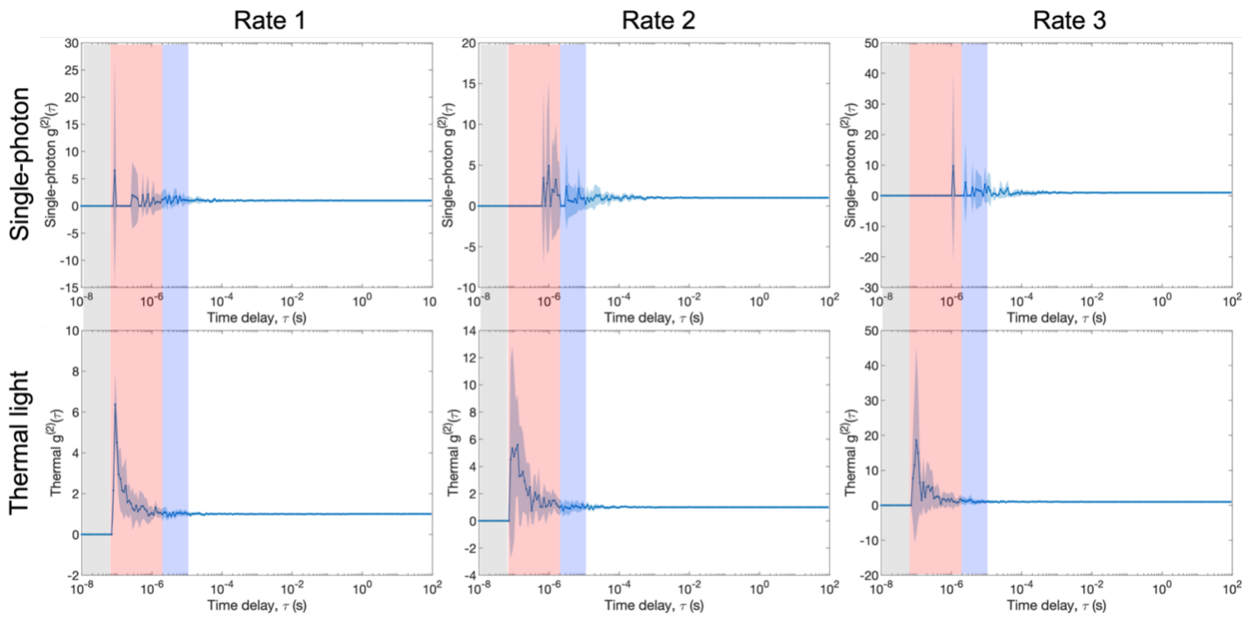

Figure S6. Second-order quantum coherence function $g^{(2)}(\tau)$, of the fluorescence light detected by Detector 2 as a function of time delay spanning from 10 ns to 100 ns in 200 time bins at Rates 1 to 3.

The second-order quantum coherence function $g^{(2)}(\tau)$ is obtained using the standard two-detector definition but employing only Detector 2, by making coincidence detections between the original Detector 2 and a delayed Detector 2 as a function of their relative time delay τ. The delayed Detector 2 is introduced by the software of the Time Tagger module; this introduces a virtually delayed channel that is treated in the same way as a real detector channel and can be used for coincidence measurement. Blue dots and solid lines are the measured data averaged by 10 repeated data recordings, each made with a 200-s integration time. The blue shading sandwiching the data points and connecting lines marks the standard deviation of the 10 repeats. The gray shaded region marks the region where $g^{(2)}(\tau) = 0$ due to the 86 ns deadtime of the detector. The red shading marks the region where $g^{(2)}(\tau)$ shows some features. For the single-photon cases, the features are essentially noise around $g^{(2)}(\tau) = 1$ given their large standard deviation. For the pseudothermal light cases, the features are suspected to arise from the significant background in the fluorescence signal, which has a time scale about few 100s of ns, being long-lived fluorescence or short-lived phosphorescence from the setup or environment. The violet shaded regions spanning 2-10 μs show no clear features given the standard deviation, which further supports that emission from the bacteriochlorophyll triplet states (lifetime $\sim$ 2-8 μs (*32*)) can be ignored in our experiments.

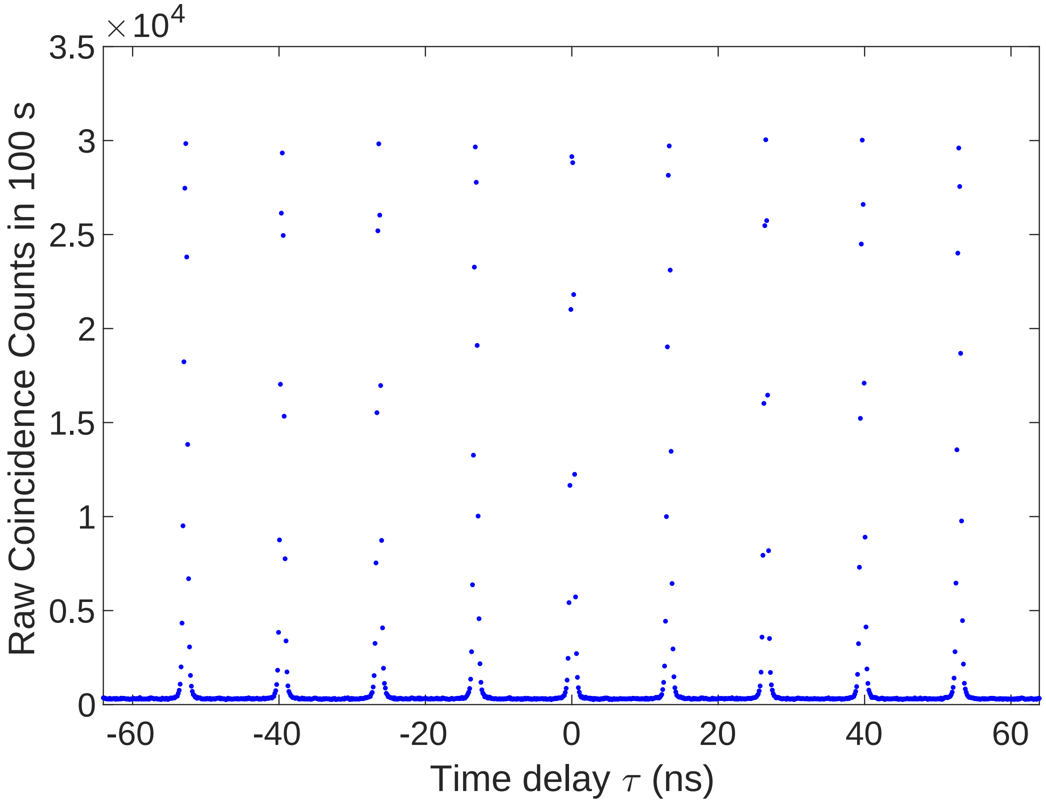

Figure S7. The raw unnormalized data of main Figure 3C.

This figure shows the measured raw unnormalized cross-correlation coincidence counts in 100-s integration time, with 128 ps bin size, of the pseudothermal incident light by standard two-detector measurements at Rate 1, ignoring the herald/gate channel. The raw coincidence count data was fit by nine equally spaced Gaussian peaks with six free parameters: the time interval between adjacent peaks, the position of the center peak, the height of the center peak, the equal height of all side peaks, the equal width of all peaks, and a constant background (see Table S5). The second-order quantum coherence function $g^{(2)}(\tau)$ in Figure 3C of the main text is normalized by the resulting equal height parameter of all side peaks.

4 Pulse-averaged $g_p^{(2)}$ of pseudothermal light generated by the SPDC

Detailed analysis of the pulse-averaged $g_p^{(2)}(\tau = 0)$ in one arm of an SPDC output in terms of the SPDC parameters is outlined in Ref. (*28*). We provide an explicit calculation for our experimental setup here, to validate our experimental measurement of $g_p^{(2)}(\tau = 0)$ for the pseudothermal incident light. In our experiment, the SPDC output is periodically pulsed. Since the repetition time between the pulses are long compared to the duration of the SPDC output pulses, the SPDC output pulses are independent of one another. In our SPDC setup, the light pulses are very weak and contain a very small number of photons. Denoting P_n as the probability of having n photons in a pulse, the weak pulse condition can be expressed as

$$P_0 \gg P_1 \gg P_2 \gg \cdots . \tag{30}$$

In this case, $g_p^{(2)}(\tau = 0)$ can be expressed in terms of P_n as

$$g_p^{(2)}(\tau = 0) = \frac{(2 \times 1)P_2 + (3 \times 2)P_3 + \cdots}{(P_1 + 2P_2 + \cdots)^2} \approx \frac{2P_2}{P_1^2}. \tag{31}$$

The state of the SPDC output is

$$\begin{aligned} |\Psi\rangle_{\text{SPDC}} &= \exp\Big(\eta \int d\omega_1 \int d\omega_2\, \psi(\omega_1, \omega_2) a^\dagger(\omega_1) b^\dagger(\omega_2)\Big)|\text{vac}\rangle \\ &= |\Psi_0\rangle + |\Psi_1\rangle + |\Psi_2\rangle + \cdots . \end{aligned} \tag{32}$$

Here $\psi(\omega_1, \omega_2)$ is the biphoton wavefunction, which is normalized as

$$\int d\omega_1 d\omega_2\, |\psi(\omega_1, \omega_2)|^2 = 1, \tag{33}$$

$a^\dagger(\omega)$ and $b^\dagger(\omega)$ denote the photon creation operators for the two output arms, and we have decomposed $|\Psi\rangle_{\text{SPDC}}$ into a vacuum part $|\Psi_0\rangle$, a single-pair part $|\Psi_1\rangle$ with one photon in mode a and one photon in mode b, and a double-pair part $|\Psi_2\rangle$ with two photons in mode a and two photons in mode b. Under weak pumping of the SPDC, $\eta \ll 1$. Expanding the exponential operator yields

$$|\Psi_1\rangle = \eta \int d\omega_1 d\omega_2\, \psi(\omega_1, \omega_2) a^\dagger(\omega_1) b^\dagger(\omega_2)|\text{vac}\rangle \tag{34}$$

and

$$|\Psi_2\rangle = \frac{\eta^2}{2} \int d\omega_1 d\omega_2 d\omega_1' d\omega_2'\, \psi(\omega_1, \omega_2)\psi(\omega_1', \omega_2') a^\dagger(\omega_1) a^\dagger(\omega_1') b^\dagger(\omega_2) b^\dagger(\omega_2')|\text{vac}\rangle. \tag{35}$$

The probability of having one photon in arm a is

$$P_1 = \langle\Psi_1|\Psi_1\rangle = \eta^2 \int d\omega_1 d\omega_2\, |\psi(\omega_1, \omega_2|^2 = \eta^2. \tag{36}$$

The probability of having two photons in arm a is

$$\begin{aligned} P_2 &= \langle\Psi_2|\Psi_2\rangle \\ &= \frac{\eta^4}{4} \int d\omega_1 d\omega_2 d\omega_1' d\omega_2' \\ &\qquad \psi^*(\omega_1, \omega_2)\psi^*(\omega_1', \omega_2')\psi(\omega_1, \omega_2)\psi(\omega_1', \omega_2') \\ &\qquad + \psi^*(\omega_1, \omega_2')\psi^*(\omega_1', \omega_2)\psi(\omega_1, \omega_2)\psi(\omega_1', \omega_2') \\ &\qquad + \psi^*(\omega_1', \omega_2)\psi^*(\omega_1, \omega_2')\psi(\omega_1, \omega_2)\psi(\omega_1', \omega_2') \\ &\qquad + \psi^*(\omega_1', \omega_2')\psi^*(\omega_1, \omega_2)\psi(\omega_1, \omega_2)\psi(\omega_1', \omega_2') \\ &= \frac{\eta^4}{2}\Big(1 + \int d\omega_1 d\omega_2 d\omega_1' d\omega_2'\, \psi^*(\omega_1, \omega_2')\psi^*(\omega_1', \omega_2)\psi(\omega_1, \omega_2)\psi(\omega_1', \omega_2')\Big). \end{aligned} \tag{37}$$

Using Eqs. (36) and (37) in Eq. (31) yields

$$g_p^{(2)}(\tau = 0) = 1 + \int d\omega_1 d\omega_2 d\omega_1' d\omega_2' \, \psi^*(\omega_1, \omega_2')\psi^*(\omega_1', \omega_2)\psi(\omega_1, \omega_2)\psi(\omega_1', \omega_2'). \tag{38}$$

We perform a Schmidt decomposition (or a singular value decomposition) on the biphoton wavefunction:

$$\psi(\omega_1, \omega_2) = \sum_i c_i \phi_i(\omega_1)\chi_i(\omega_2), \tag{39}$$

to generate the positive real number coefficients c_i. Here ϕ_i and χ_i are in general complex-valued and are normalized such that $\int d\omega |\phi_i(\omega)|^2 = \int d\omega |\chi_i(\omega)|^2 = 1$. An important property of the Schmidt decomposition is that $\phi(\omega)$ and $\chi(\omega)$ satisfy the orthogonal relations: $\int d\omega \phi_i^*(\omega)\phi_j(\omega) = \int d\omega \chi_i^*(\omega)\chi_j(\omega) = \delta_{ij}$. Note that the normalization of $\psi(\omega_1, \omega_2)$ (Eq. (33)) implies that $\sum_i c_i^2 = 1$. Substituting Eq. (39) into Eq. (38) and using the orthogonality relations results in an explicit multi-mode equation for $g_p^{(2)}(\tau = 0)$,

$$g_p^{(2)}(\tau = 0) = 1 + \sum_i c_i^4, \tag{40}$$

which can be readily calculated numerically given a biphoton wavefunction, to determine the number of contributing modes.

5 Modeling the biphoton wavefunction

For SPDC pumped by a classical laser pulse, the biphoton wavefunction is modeled as

$$\psi(\omega_i, \omega_s) = \mathcal{N} \times \text{PEF}(\omega_i, \omega_s) \times \text{PMF}(\omega_i, \omega_s), \tag{41}$$

a product of a pulse envelope function PEF and a phase matching function PMF (*55-56*). ω_i and ω_s are the frequencies of the idler and signal photons, respectively, and $\mathcal{N}$ is a normalization factor. The PEF is defined as

$$\text{PEF}(\omega_i, \omega_s) = \xi(\omega_i + \omega_s), \tag{42}$$

where $\xi(\omega)$ is the frequency profile of the pump pulse amplitude. This enforces the energy conservation in SPDC, such that the sum of the energy of the two photons is equal to the energy of the pump pulse. The PMF is defined as (*55*)

$$\text{PMF}(\omega_i, \omega_s) = \sinh(\frac{\Delta k L}{2}), \tag{43}$$

where L is the length of the SPDC crystal and Δk is specified by

$$\Delta k = k_p(\omega_i + \omega_s) - k_i(\omega_i) - k_s(\omega_s) - \frac{2\pi}{\Lambda}. \tag{44}$$

Here $k_p(\omega_i + \omega_s)$, $k_i(\omega_i)$, and $k_s(\omega_s)$ are the wavevectors of the pump pulse, the idler photon, and the signal photon at frequencies $\omega_i + \omega_s$, ω_i, and ω_s, respectively. Λ is the poling period of the crystal. The phase matching function PMF enforces momentum conservation. If the crystal length is infinite, the momentum would be exactly conserved due to the continuous translational symmetry. However, since the crystal has a finite length, momentum is not exactly conserved and $\Delta k L$ characterizes the deviation from exact momentum conservation. We use the full dispersion relation $\omega(k)$ from the database (*57-58*) to model the biphoton wavefunction in our numerical calculations.

5.1 Pump pulse frequency profile

The intensity spectrum of the pump pulse is shown in Figure S8. To calibrate the raw spectrum such that the center wavelength is at 404 nm, we have shifted the wavelength lower by 5.354 nm from the raw data. The spectrum was fitted with both a quadratic exponential function (i.e., a Gaussian function) and a quartic

exponential function. We take the quartic exponential function as the pump intensity spectrum, since it produces a better fit to the experimental spectrum. The resulting intensity spectrum is

$$I(\omega) \propto e^{-b(\omega-\omega_0)^4}, \tag{45}$$

with fitting parameter b equal to $b = 6.158\times10^{-7}\,\mathrm{ps}^4$. The center frequency $\omega_0 = 4662.6\,\mathrm{ps}^{-1}$ is the frequency corresponding to 404 nm wavelength.

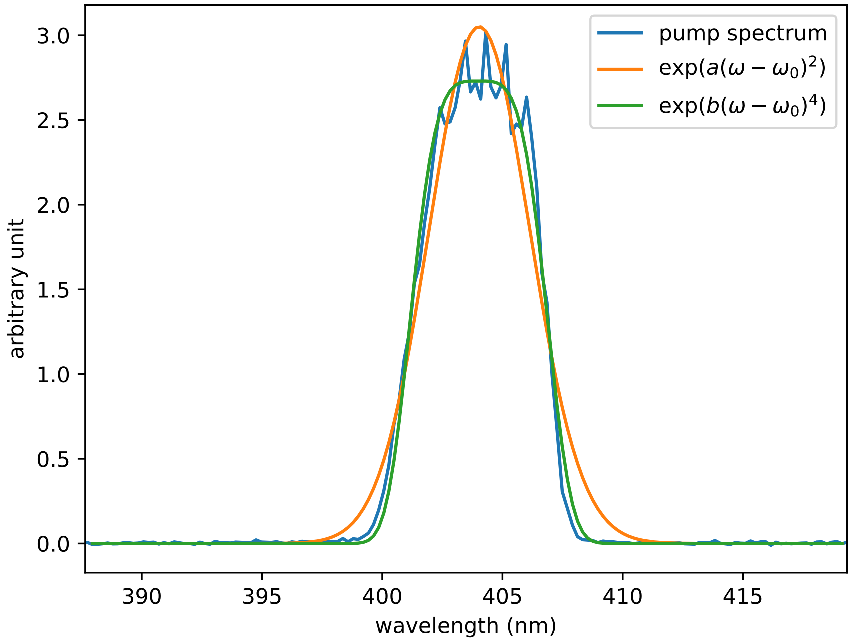

Figure S8. Intensity spectrum of the pump pulse. The spectrum is fitted with a quadratic exponential and a quartic exponential function which gives a better fit.

We take the electric field frequency profile $\xi_0(\omega)$ to be proportional to the square root of the intensity frequency profile $I(\omega)$, i.e.,

$$\xi_0(\omega) = e^{-\frac{b}{2}(\omega-\omega_0)^4}. \tag{46}$$

The pump pulse propagates through a 2 m SiO_2 optical fiber. To account for the pulse stretching effect due to group velocity dispersion in the fiber, we write the frequency profile after the fiber as

$$\tilde{\xi}_1(\omega) = \xi_0(\omega)e^{ik(\omega)L}, \tag{47}$$

where $L = 2\,\mathrm{m}$ is the length of the fiber. $k(\omega)$ is the dispersion that relates the wave vector k to the frequency ω in quartz (SiO_2). The dispersion relation is obtained from the database (*57-58*). The dispersion relation can be Taylor expanded around the central frequency ω_0 as

$$k(\omega) = k(\omega_0) + \frac{1}{v_g(\omega_0)}(\omega-\omega_0) + \cdots . \tag{48}$$

The zeroth order term represents a constant phase factor and the first order term represents the time translation of the pulse. Pulse stretching effects are due to higher-order expansion terms. To focus on the pulse stretching effect, we therefore remove the zeroth and first order terms and take the frequency profile after the fiber as

$$\xi_1(\omega) = \xi_0(\omega)\exp\Big(iL\big(k(\omega) - k(\omega_0) - \frac{1}{v_g(\omega_0)}(\omega-\omega_0)\big)\Big). \tag{49}$$

Figure S9 shows the effect of pulse stretching due to the optical fiber, in the time domain. Before entering the fiber, the pulse has a duration of around 0.2 ps. After exiting the fiber, the pulse duration is stretched to around 20 ps. Eq. (49) is then used to evaluate the PEF in Eq. (42).

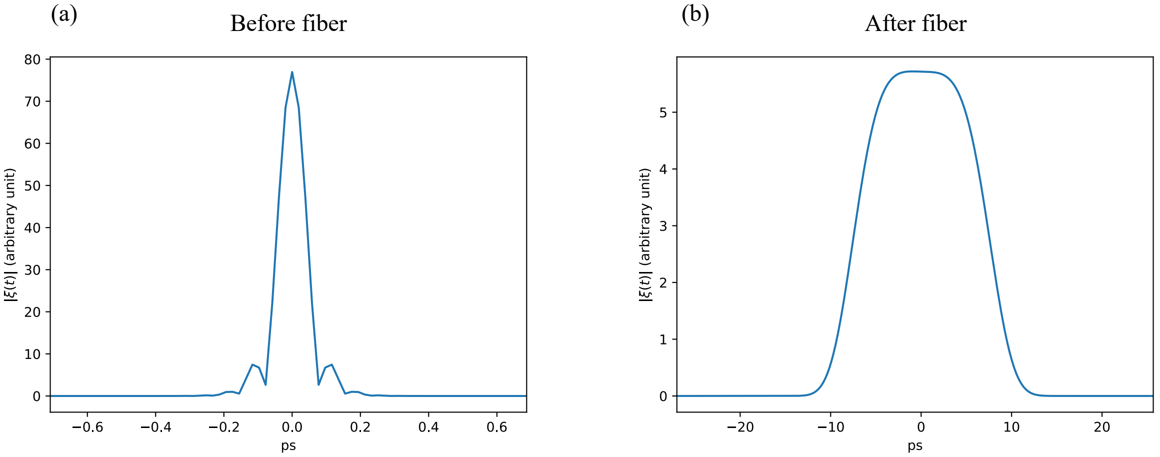

Figure S9. The pump pulse in the time domain. (a) Before entering the fiber, (b) after exiting the fiber. Due to dispersion effects the pulse duration is stretched from ~ 0.2 ps to ~ 20 ps, resulting in an approximately 100-fold increase in pulse duration.

5.2 Modeling the phase matching function (PMF)

A 30 mm long periodically poled potassium titanyl phosphate (PPKTP) crystal (poling period 9.825 um) is used as the SPDC medium. The pump pulse and the idler photon are polarized in the y direction (Extraordinary beam). The signal photon is polarized in the z direction (Ordinary beam). To model the PMF, we use the dispersion relation for $KTiOPO_4$ in the database (*58-59*). The PMF is then calculated using Eqs. (43).

5.3 Biphoton wavefunction in the time domain.

The biphoton wavefunction $\psi(\omega_i, \omega_s)$ is the product of the PEF and the PMF (see Eq. (41)). Figure S10 shows the amplitudes of the PEF (panel (a)), the PMF (panel (b)) and $|\psi(\omega_i, \omega_s|$, and Figure S11 (a) shows a zoomed-in view of $|\psi(\omega_i, \omega_s)|$. The shape of the PEF extends along the anti-diagonal because it is a function of $\omega_i + \omega_s$ and therefore stretches along the $\omega_i + \omega_s$ direction (Section 5.1). The shape of the PMF is slanted away from the anti-diagonal due to the different dispersion relations along the y and z directions in the crystal. Since the shapes of PEF and PMF are not parallel, they have a finite overlap region, and the biphoton wavefunction $\psi(\omega_i, \omega_s)$ has significant amplitude only in a finite frequency range. Applying Eqs. (39) and (40), we find $g_p^{(2)} = 1.001$, consistent with the measured experimental value of $g^{(2)}(\tau = 0) = 1.0103 \pm 0.0104$ given in the main text. Figure S11 (b) plots the singular values in descending order. By visual inspection, there are around 1000 modes that contribute significantly to the biphoton wavefunction. Figure S11 (c) shows the amplitude of the biphoton wavefunction in the time domain, i.e., $|\psi(t_i, t_s)|$, where

$$\psi(t_i, t_s) = \int d\omega_i \int d\omega_s \, e^{-i\omega_i t_i} e^{-i\omega_s t_s} \psi(\omega_i, \omega_s). \tag{50}$$

As expected, the time domain biphoton wavefunction also has a wide spread in the diagonal direction, complementary to the narrow spread of the frequency domain ψ in this direction and reflecting the presence of multiple temporal modes in the quantum state. Similarly, the time domain ψ has a narrow spread in the anti-diagonal direction, while the frequency domain ψ has a wide spread in the anti-diagonal direction.

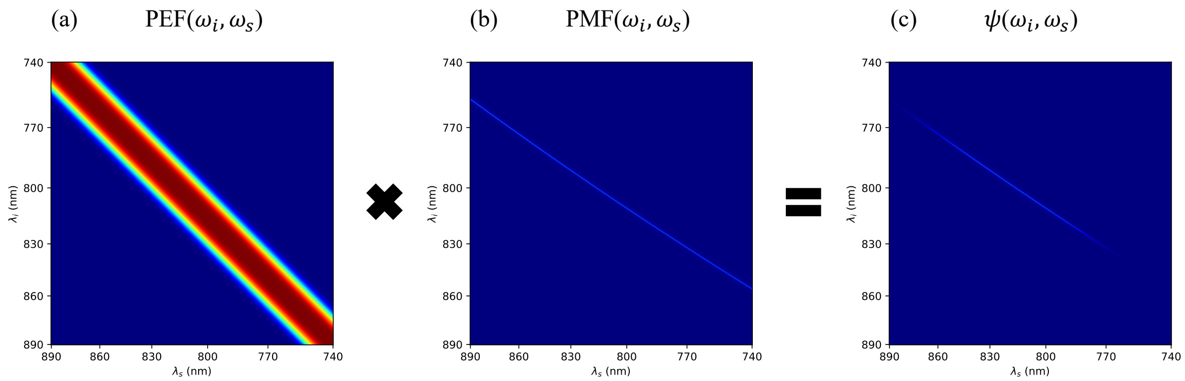

Figure S10. Characteristics of the biphoton wavefunction $\psi(\omega_i, \omega_s)$. (a) Amplitude of the pulse envelope function, $|\text{PEF}(\omega_i, \omega_s)|$. (b) Amplitude of the phase matching function, $|\text{PMF}(\omega_i, \omega_s)|$. (c) Amplitude of the biphoton wavefunction, $|\psi(\omega_i, \omega_s)|$. The scales of the axes are linear in frequency, and the corresponding wavelengths are labeled on the axes. The biphoton wavefunction is the product of the PEF and the PMF (see Eq. (41)). $|\psi(\omega_i, \omega_s)|$ is clearly stretched in the $\omega_i - \omega_s$ direction and squeezed in the $\omega_i + \omega_s$ direction, resulting in strong anticorrelation between ω_i and ω_s.

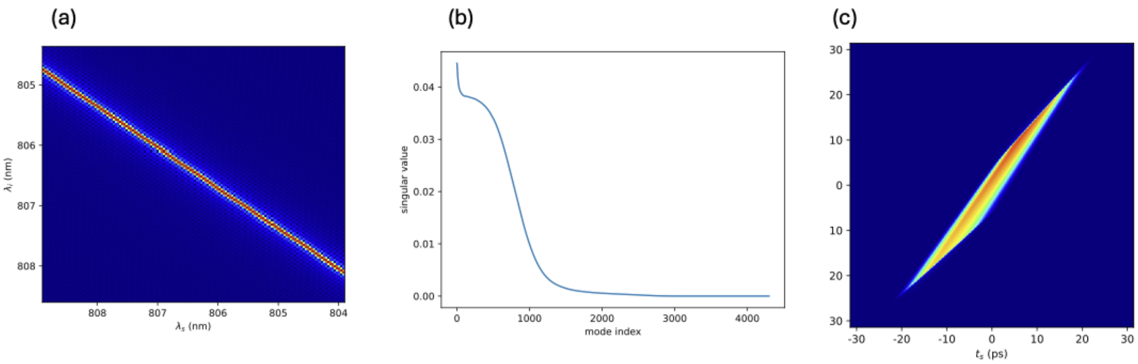

Figure S11. (a) Zoomed-in view of the amplitude of the biphoton wavefunction, $|\psi(\omega_i, \omega_s)|$. (b) Singular values of the biphoton wavefunction $\psi(\omega_i, \omega_s)$. Visual inspection shows that ~ 1000 modes contribute significantly to ψ. (c) Amplitude of the biphoton wavefunction in the time domain, $|\psi(t_i, t_s)|$. This shows a high degree of correlation that is complementary to the anti-correlation of the frequency domain representation in Figure S10.

REFERENCES AND NOTES

1. A. Einstein, Über einen die Erzeugung und Verwandlung des Lichtes betreffenden heuristischen Gesichtspunkt. *Ann. Phys.* **322**, 132–148 (1905). [On a heuristic perspective of the generation and transformation of light].

2. R. H. Brown, R. Q. Twiss, Correlation between photons in two coherent beams of light. *Nature* **177**, 27–29 (1956).

3. R. J. Glauber, The quantum theory of optical coherence. *Phys. Rev.* **130**, 2529–2539 (1963).

4. R. Loudon, *The Quantum Theory of Light* (Oxford Univ. Press, ed. 3, 2000).

5. C. Gerry, P. Knight, *Introductory Quantum Optics* (Cambridge Univ. Press, 2012).

6. R. E. Blankenship, *Molecular Mechanisms of Photosynthesis* (John Wiley & Sons, 2014).

7. S. Savikhin, W. S. Struve, Temperature dependence of electronic energy transfers within B850 antennae of the NF57 mutant of the purple bacterium *Rhodobacter sphaeroides*. *Chem. Phys.* **210**, 91–100 (1996).

8. H. van Amerongen, R. van Grondelle, L. Valkunas, *Photosynthetic Excitons* (World Scientific, 2000).

9. T. Brixner, J. Stenger, H. M. Vaswani, M. Cho, R. E. Blankenship, G. R. Fleming, Two-dimensional spectroscopy of electronic couplings in photosynthesis. *Nature* **434**, 625–628 (2005).

10. G. S. Engel, T. R. Calhoun, E. L. Read, T.-K. Ahn, T. Mančal, Y.-C. Cheng, R. E. Blankenship, G. R. Fleming, Evidence for wavelike energy transfer through quantum coherence in photosynthetic systems. *Nature* **446**, 782–786 (2007).

11. G. Panitchayangkoon, D. Hayes, K. A. Fransted, J. R. Caram, E. Harel, J. Wen, R. E. Blankenship, G. S. Engel, Long-lived quantum coherence in photosynthetic complexes at physiological temperature. *Proc. Natl. Acad. Sci. U.S.A.* **107**, 12766–12770 (2010).

12. G. D. Scholes, G. R. Fleming, A. Olaya-Castro, R. van Grondelle, Lessons from nature about solar light harvesting. *Nat. Chem.* **3**, 763–774 (2011).

13. V. Butkus, D. Zigmantas, L. Valkunas, D. Abramavicius, Vibrational vs. electronic coherences in 2D spectrum of molecular systems. *Chem. Phys. Lett.* **545**, 40–43 (2012).

14. A. Chenu, G. D. Scholes, Coherence in energy transfer and photosynthesis. *Annu. Rev. Phys. Chem.* **66**, 69–96 (2015).

15. G. D. Scholes, G. R. Fleming, L. X. Chen, A. Aspuru-Guzik, A. Buchleitner, D. F. Coker, G. S. Engel, R. van Grondelle, A. Ishizaki, D. M. Jonas, J. S. Lundeen, J. K. McCusker, S. Mukamel, J. P. Ogilvie, A. Olaya-Castro, M. A. Ratner, F. C. Spano, K. B. Whaley, X. Zhu, Using coherence to enhance function in chemical and biophysical systems. *Nature* **543**, 647–656 (2017).

16. R. Croce, R. van Grondelle, H. van Amerongen, I van Stokkum, Eds. *Light Harvesting in Photosynthesis* (Routledge & CRC Press, 2021). https://routledge.com/Light-Harvesting-in-Photosynthesis/Croce-Grondelle-Amerongen-Stokkum/p/book/9780367781491.

17. D. M. Jonas, Vibrational and nonadiabatic coherence in 2D electronic spectroscopy, the Jahn–Teller effect, and energy transfer. *Annu. Rev. Phys. Chem.* **69**, 327–352 (2018).

18. E. Thyrhaug, R. Tempelaar, M. J. P. Alcocer, K. Žídek, D. Bína, J. Knoester, T. L. C. Jansen, D. Zigmantas, Identification and characterization of diverse coherences in the Fenna–Matthews–Olson complex. *Nat. Chem.* **10**, 780–786 (2018).

19. J. Cao, R. J. Cogdell, D. F. Coker, H.-G. Duan, J. Hauer, U. Kleinekathöfer, T. L. C. Jansen, T. Mančal, R. J. D. Miller, J. P. Ogilvie, V. I. Prokhorenko, T. Renger, H.-S. Tan, R. Tempelaar, M. Thorwart, E. Thyrhaug, S. Westenhoff, D. Zigmantas, Quantum biology revisited. *Sci. Adv.* **6**, eaaz4888 (2020).

20. E. A. Arsenault, Y. Yoneda, M. Iwai, K. K. Niyogi, G. R. Fleming, Vibronic mixing enables ultrafast energy flow in light-harvesting complex II. *Nat. Commun.* **11**, 1460 (2020).

21. T. Mančal, L. Valkunas, Exciton dynamics in photosynthetic complexes: Excitation by coherent and incoherent light. *New J. Phys.* **12**, 065044 (2010).

22. A. Chenu, A. M. Brańczyk, G. D. Scholes, J. E. Sipe, Thermal light cannot be represented as a statistical mixture of single pulses. *Phys. Rev. Lett.* **114**, 213601 (2015).

23. P. Brumer, Shedding (incoherent) light on quantum effects in light-induced biological processes. *J. Phys. Chem. Lett.* **9**, 2946–2955 (2018).

24. Q. Li, K. Orcutt, R. L. Cook, J. Sabines-Chesterking, A. L. Tong, G. S. Schlau-Cohen, X. Zhang, G. R. Fleming, K. B. Whaley, Single-photon absorption and emission from a natural photosynthetic complex. *Nature* **619**, 300–304 (2023).

25. P. G. Kwiat, K. Mattle, H. Weinfurter, A. Zeilinger, A. V. Sergienko, Y. Shih, New high-intensity source of polarization-entangled photon pairs. *Phys. Rev. Lett.* **75**, 4337–4341 (1995).

26. P. Grangier, G. Roger, A. Aspect, Experimental evidence for a photon anticorrelation effect on a beam splitter: A new light on single-photon interferences. *Europhys. Lett.* **1**, 173–179 (1986).

27. N. Piro, F. Rohde, C. Schuck, M. Almendros, J. Huwer, J. Ghosh, A. Haase, M. Hennrich, F. Dubin, J. Eschner, Heralded single-photon absorption by a single atom. *Nat. Phys.* **7**, 17–20 (2011).

28. Z.-Y. J. Ou, *Multi-Photon Quantum Interference* (Springer, 2007).

29. Y. Fujihashi, R. Shimizu, A. Ishizaki, Generation of pseudo-sunlight via quantum entangled photons and the interaction with molecules. *Phys. Rev. Research* **2**, 023256 (2020).

30. N. Quesada, A. M. Brańczyk, Broadband pseudothermal states with tunable spectral coherence generated via nonlinear optics. *Phys. Rev. A* **99**, 013830 (2019).

31. D. Iluz, Z. Dubinsky, “Quantum yields in aquatic photosynthesis,” in *Photosynthesis*, Z. Dubinsky, Ed. (InTech, 2013); http://intechopen.com/books/photosynthesis/quantum-yields-in-aquatic-photosynthesis.

32. T. G. Monger, R. J. Cogdell, W. W. Parson, Triplet states of bacteriochlorophyll and carotenoids in chromatophores of photosynthetic bacteria. *Biochim. Biophys. Acta* **449**, 136–153 (1976).

33. M. Beck, Comparing measurements of $g^{(2)}(0)$ performed with different coincidence detection techniques. *J. Opt. Soc. Am. B* **24**, 2972 (2007).

34. P. R. Tapster, J. G. Rarity, Photon statistics of pulsed parametric light. *J. Mod. Opt.* **45**, 595–604 (1998).

35. B. E. A. Saleh, M. C. Teich, *Fundamentals of Photonics* (Wiley, ed. 3, 2019).

36. M. Even Tzur, M. Birk, A. Gorlach, M. Krüger, I. Kaminer, O. Cohen, Photon-statistics force in ultrafast electron dynamics. *Nat. Photonics* **17**, 501–509 (2023).

37. H. Yang, G. Luo, P. Karnchanaphanurach, T.-M. Louie, I. Rech, S. Cova, L. Xun, X. S. Xie, Protein conformational dynamics probed by single-molecule electron transfer. *Science* **302**, 262–266 (2003).

38. M. Pelton, G. Smith, N. F. Scherer, R. A. Marcus, Evidence for a diffusion-controlled mechanism for fluorescence blinking of colloidal quantum dots. *Proc. Natl. Acad. Sci. U.S.A.* **104**, 14249–14254 (2007).

39. A. L. Efros, D. J. Nesbitt, Origin and control of blinking in quantum dots. *Nat. Nanotechnol.* **11**, 661–671 (2016).

40. C. Bradac, M. T. Johnsson, M. V. Breugel, B. Q. Baragiola, R. Martin, M. L. Juan, G. K. Brennen, T. Volz, Room-temperature spontaneous superradiance from single diamond nanocrystals. *Nat. Commun.* **8**, 1205 (2017).

41. E. Wientjes, J. Renger, A. G. Curto, R. Cogdell, N. F. van Hulst, Strong antenna-enhanced fluorescence of a single light-harvesting complex shows photon antibunching. *Nat. Commun.* **5**, 4236 (2014).

42. M. Ketelaars, A. M. Van Oijen, M. Matsushita, J. Köhler, J. Schmidt, T. J. Aartsma, Spectroscopy on the B850 band of individual light-harvesting 2 complexes of *Rhodopseudomonas acidophila* I. Experiments and Monte Carlo simulations. *Biophys. J.* **80**, 1591–1603 (2001).

43. N. R. S. Reddy, R. Picorel, G. J. Small, B896 and B870 components of the *Rhodobacter sphaeroides* antenna: A hole burning study. *J. Phys. Chem.* **96**, 6458–6464 (1992).

44. J. Kim, T. C. Nguyen-Phan, A. T. Gardiner, T. H. Yoon, R. J. Cogdell, M. Cho, G. D. Scholes, Vibrational modes promoting exciton relaxation in the B850 band of LH2. *J. Phys. Chem. Lett.* **13**, 1099–1106 (2022).

45. D. I. H. Holdaway, V. Notararigo, A. Olaya-Castro, Perturbation approach for computing frequency- and time-resolved photon correlation functions. *Phys. Rev. A* **98**, 063828 (2018).

46. C. Sánchez Muñoz, F. Schlawin, Photon correlation spectroscopy as a witness for quantum coherence. *Phys. Rev. Lett.* **124**, 203601 (2020).

47. C. Nation, V. Notararigo, H. O. Gestsson, L. Sapienza, A. Olaya-Castro, Two-colour photon correlations probe coherent vibronic contributions to electronic excitation transport under incoherent illumination. arXiv:2403.00857 [physics.chem-ph] (2024).

48. C. Nation, H. O. Gestsson, A. Olaya-Castro, Photon correlation time-asymmetry and dynamical coherence in multichromophoric systems. arXiv:2404.16892 [physics.chem-ph] (2024).

49. C. Hofmann, T. J. Aartsma, J. Köhler, Energetic disorder and the B850-exciton states of individual light-harvesting 2 complexes from *Rhodopseudomonas acidophila*. *Chem. Phys. Lett.* **395**, 373–378 (2004).

50. M. Grimaldo, F. Roosen-Runge, F. Zhang, F. Schreiber, T. Seydel, Dynamics of proteins in solution. *Q. Rev. Biophys.* **52**, e7 (2019).

51. H. C. H. Chan, O. E. Gamel, G. R. Fleming, K. B. Whaley, Single-photon absorption by single photosynthetic light-harvesting complexes. *J. Phys. B At. Mol. Opt. Phys.* **51**, 054002 (2018).

52. L. Ko, R. L. Cook, K. B. Whaley, Dynamics of photosynthetic light harvesting systems interacting with N-photon Fock states. *J. Chem. Phys.* **156**, 244108 (2022).

53. A. L. Tong, O. C. Fiebig, M. Nairat, D. Harris, M. Giansily, A. Chenu, J. N. Sturgis, G. S. Schlau-Cohen, Comparison of the energy-transfer rates in structural and spectral variants of the B800–850 complex from purple bacteria. *J. Phys. Chem. B.* **124**, 1460–1469 (2020).

54. J. I. Ogren, A. L. Tong, S. C. Gordon, A. Chenu, Y. Lu, R. E. Blankenship, J. Cao, G. S. Schlau-Cohen, Impact of the lipid bilayer on energy transfer kinetics in the photosynthetic protein LH2. *Chem. Sci.* **9**, 3095–3104 (2018).

55. C. Zhang, Y. Huang, B. Liu, C. Li, G. Guo, Spontaneous parametric down-conversion sources for multiphoton experiments. *Adv. Quantum Technol.* **4**, 2000132 (2021).

56. J. J. Miguel Varga, J. Lasa-Alonso, M. Molezuelas-Ferreras, N. Tischler, G. Molina-Terriza, Bandwidth control of the biphoton wavefunction exploiting spatio-temporal correlations. *Opt. Commun.* **504**, 127461 (2022).

57. G. Ghosh, Dispersion-equation coefficients for the refractive index and birefringence of calcite and quartz crystals. *Opt. Commun.* **163**, 95–102 (1999).

58. M. N. Polyanskiy, Refractiveindex.info database of optical constants. *Sci. Data* **11**, 94 (2024).

59. K. Kato, E. Takaoka, Sellmeier and thermo-optic dispersion formulas for ktp. *Appl. Optics* **41**, 5040–5044 (2002).
